# Selective production of arenes via direct lignin upgrading over a niobium-based catalyst

**DOI:** 10.1038/ncomms16104

**Published:** 2017-07-24

**Authors:** Yi Shao, Qineng Xia, Lin Dong, Xiaohui Liu, Xue Han, Stewart F. Parker, Yongqiang Cheng, Luke L. Daemen, Anibal J. Ramirez-Cuesta, Sihai Yang, Yanqin Wang

**Affiliations:** 1 Shanghai Key Laboratory of Functional Materials Chemistry, Research Institute of Industrial Catalysis, School of Chemistry and Molecular Engineering, East China University of Science and Technology, Shanghai 200237, China; 2 School of Chemistry, University of Manchester, Manchester M13 9PL, UK; 3 ISIS Facility, STFC Rutherford Appleton Laboratory, Chilton, Oxfordshire OX11 0QX, UK; 4 The Chemical and Engineering Materials Division (CEMD), Neutron Sciences Directorate, Oak Ridge National Laboratory, Oak Ridge, Tennessee 37831, USA

## Abstract

Lignin is the only large-volume renewable source of aromatic chemicals. Efficient depolymerization and deoxygenation of lignin while retaining the aromatic functionality are attractive but extremely challenging. Here we report the selective production of arenes via direct hydrodeoxygenation of organosolv lignin over a porous Ru/Nb_2_O_5_ catalyst that enabled the complete removal of the oxygen content from lignin. The conversion of birch lignin to monomer C_7_–C_9_ hydrocarbons is nearly quantitative based on its monomer content, with a total mass yield of 35.5 wt% and an exceptional arene selectivity of 71 wt%. Inelastic neutron scattering and DFT calculations confirm that the Nb_2_O_5_ support is catalytically unique compared with other traditional oxide supports, and the disassociation energy of *C*_aromatic_–OH bonds in phenolics is significantly reduced upon adsorption on Nb_2_O_5_, resulting in its distinct selectivity to arenes. This one-pot process provides a promising approach for improved lignin valorization with general applicability.

## Abstract (web summary)

Lignin is cheap and abundant, and potentially a major source of aromatic compounds. Here the authors show selective production of arenes via hydrodeoxygenation of lignin over Ru/Nb_2_O_5_, and mechanistically show the strong influence of the Nb_2_O_5_ support on phenolic bond dissociation energies.

Lignin is a complex aromatic biopolymer that makes up 25–35% of renewable carbon in the world^1^, and is the only large-volume renewable source of aromatic chemicals^2^. The extended network of lignin is constructed from monomers of coumaryl, coniferyl and sinapyl alcohols via intermolecular C–O and C–C bonds in a random order (Fig. 1)^3^,^4^,^5^,^6^. Because of its highly irregular polymeric structure and recalcitrant nature, lignin is widely regarded as a waste product in industry, and traditionally combusted to generate heat and power. Production of feedstocks with higher value via lignin catalytic conversion has been recognized as an emerging approach to bridge future gaps in the supply of aromatic chemicals^3^,^4^,^5^,^6^.

Recently, a catalytic hydropyrolysis process has been developed to convert lignin into aromatic compounds over a catalyst of H-USY (yield: ∼30 wt%) or Pd/HZSM-5 (yield: ∼44 wt%) at 650 °C (refs 7, 8). Although effective, pyrolysis is generally very energy consuming^4^,^5^,^9^. An alternative strategy for lignin valorization is based on the depolymerization of lignin into low-molecular-weight feedstocks followed by sequential upgrading to form useful chemicals and fuels^4^,^5^. The first depolymerization step is a major impediment; multiple strategies are reported to address this challenge, such as wet oxidation processes to give aromatic aldehydes and acids^10^,^11^, depolymerization of selectively oxidized lignin into syringyl and guaiacyl-derived ketones^12^,^13^ and, more recently, an efficient formaldehyde-assisted lignin depolymerization into various guaiacyl and syringyl monomers^14^. Additional strategies based on hydrogen reduction^15^ and depolymerizations in supercritical^16^,^17^,^18^ or conventional alcohols^19^,^20^ have also been proposed. However, products obtained from all the above approaches are primarily oxygen-containing compounds with a wide distribution (for example, substituted phenols, guaiacols, syringols, alcohols and ethers generated from alcohol solvents), and therefore sequential hydrogenation processes are required to convert them into hydrocarbons.

The direct conversion of lignin into low-boiling-point hydrocarbons instead of high-boiling-point phenolic compounds could greatly facilitate the lignin valorization via easier separation based on conventional refineries. Although lignin conversion into cycloalkanes as liquid fuels through deep hydrodeoxygenation reactions is a promising pathway, the aromatic functionality of lignin is completely lost accompanied with the penalty of additional H_2_ consumption^15^,^21^,^22^. Preserving the aromatic rings while selectively cleaving the C–O bonds in phenols is a highly challenging task because the C–O bond in aryl ethers is strong, especially for that in phenol (414 kJ mol^−1^). There is therefore a well-known competition between the hydrogenolysis of C–O bonds and the hydrogenation of the aromatic rings during the lignin hydrodeoxygenation^23^,^24^,^25^,^26^. Unfortunately, the latter is thermodynamically favoured and, as a result, selective production of arenes from lignin upgrading is rarely reported^27^. Design of new functional catalysts with unique selectivity is the key to overcome the barrier. Recently, niobium-based catalysts have shown great promise in promoting the cleavage of C–O bonds^28^,^29^,^30^.

Here we report a one-pot lignin conversion over a Ru/Nb_2_O_5_ catalyst in water to directly produce liquid hydrocarbons with a mass yield of 35.5 wt% and a near-quantitative carbon yield based on lignin monomers. Significantly, this process combines the depolymerization of raw lignin and the hydrogenolysis of depolymerized compounds, and affords an exceptional selectivity of 71 wt% for monomer arene. A combined inelastic neutron scattering (INS) and density functional theory (DFT) calculation analysis unambiguously confirmed the unique activity of the Nb_2_O_5_ species, thus rationalizing the observed high reactivity and unusual selectivity for Ru/Nb_2_O_5_. We found that it is a combination of strong adsorption, selective activation of the *C*_aromatic_–OH bonds in phenolics and a synergistic effect between the Ru and NbO_x_ species that results in the high selectivity to yield arenes.

## Results

### Direct hydrodeoxygenation of lignin to hydrocarbons

The hydrodeoxygenation of birch lignin (0.1 g) was conducted at 250 °C with 0.7 MPa H_2_ for 20 h over a porous Ru/Nb_2_O_5_ catalyst (0.2 g, Brunner-Emmet-Teller method (BET) surface area=54 m^2^ g^−1^) in aqueous solution. Mixtures of hydrocarbons (that is, arenes and alkanes) were produced via complete removal of oxygen functional groups from lignin (Table 1). Specifically, the reaction over the Ru/Nb_2_O_5_ catalyst reached a total mass yield of 35.5 wt% for liquid hydrocarbons, with 29.7 wt% being C_7_–C_9_ hydrocarbons. Importantly, the corresponding carbon yield of C_7_–C_9_ hydrocarbons is nearly quantitative, as evidenced from an established lignin monomer analysis based on NBO method (Supplementary Fig. 1)^14^,^31^. The detailed product distribution and yields show a significant total arene mass yield of 22.7 wt% (Table 1 and Supplementary Table 1), leading to an exceptional arene selectivity of 71 wt% for the total C_7_–C_9_ hydrocarbons.

To detect the reaction intermediates, the reaction was repeated but with a reduced time of 10 h (Supplementary Table 2). Four phenolic monomers of guaiacol and syringol derivatives (total yield of 7.7 wt%) were found together with products of arene and cycloalkane-based hydrocarbons (total yield of 9.7 wt%). This suggests that the lignin was first depolymerized to phenolic monomers via multiple steps of C–O hydrogenolysis to cleave C–O–C ether bonds, and the resultant phenolic monomers were sequentially converted to arenes and cycloalkanes by further hydrogenolysis over Ru/Nb_2_O_5_. Although depolymerization of model compounds of lignin over homogeneous or heterogeneous catalysts is widely reported to generate phenols by the cleavage of aromatic C–O bonds in aryl ethers^23^,^24^,^25^,^26^, further hydrogenolysis of the *C*_aromatic_–OH bond in phenols to produce fully deoxygenated hydrocarbons is problematic and hardly achieved. Interestingly, the Ru/Nb_2_O_5_ catalyst integrated the depolymerization and hydrogenolysis steps in lignin conversion to selectively produce arenes.

To confirm the unique activity of Ru/Nb_2_O_5_, a wide range of supported Ru catalysts (Ru/ZrO_2_, Ru/Al_2_O_3_, Ru/TiO_2_, Ru/H-ZSM-5 and Ru/C) were tested for this reaction under the same conditions (Table 1 and Supplementary Table 3), where both the total yield of C_7_–C_9_ hydrocarbons and the arene selectivity decreased dramatically in all cases. For example, over the Ru/ZrO_2_ catalyst, the mass yield of C_7_–C_9_ hydrocarbons was 18.3 wt% with 1.7 wt% phenolic monomers, and the percentage of arenes was 31 wt%, much lower than that of Ru/Nb_2_O_5_ (71 wt%). Similar results were obtained for the Ru/Al_2_O_3_ catalyst. Over the Ru/C catalyst, no C_7_–C_9_ arenes was detected, and lignin monomers were completely saturated with a 29.3 wt% yield of C_7_–C_9_ cycloalkanes. The Ru/TiO_2_ and Ru/H-ZSM-5 catalysts show even poorer activity for this reaction. To rule out the possibility that Nb_2_O_5_ functioned as a co-catalyst, Nb_2_O_5_ was mechanically blended with Ru/TiO_2_ and little change was observed in reaction yield/selectivity compared with the Ru/TiO_2_ catalyst, indicating that the synergism between the Ru and Nb_2_O_5_ species plays a key role. The blank experiment without any catalyst yielded only 1.3 wt% phenolic monomers, confirming the negligible effect of the near-critical water on this reaction. These results confirm that the Ru/Nb_2_O_5_ catalyst possesses exceptional activity for the simultaneous depolymerization and hydrogenolysis of raw lignin via the effective cleavage of C–O–C bonds in the lignin network and the selective cleavage of *C*_aromatic_–OH bonds in phenolic compounds.

The dependence of the substrate/catalyst ratio on the product distribution was also investigated (Supplementary Table 4). With reduced amount of the catalyst of 0.1 and 0.05 g (the amount of lignin remained the same), the yield of C_7_–C_9_ hydrocarbons decreased to 13.2 and 4.3 wt% with 0.2 and 8.9 wt% phenolic monomers, respectively. This decreased yield is likely due to a reduced catalyst/lignin contact since the lignin is insoluble in water. Similar observation was also reported in lignin hydropyrolysis that employed a substrate/catalyst ratio of 1/20 (ref. 9). Nonetheless, the ratios of C_7_–C_9_ arenes/C_7_–C_9_ hydrocarbons for all the reactions over the Ru/Nb_2_O_5_ catalyst were constant at 71 wt% and therefore the arene selectivity is independent to the substrate/catalyst ratio, indicating that the nature of the lignin/catalyst interaction remains the same regardless of the substrate/catalyst ratio.

Upgrading of lignin extracted from beech and pine was also conducted over the Ru/Nb_2_O_5_ catalyst with both high total mass yields of liquid hydrocarbons and high arene selectivities achieved (Supplementary Table 5). This result confirms the general applicability of the Ru/Nb_2_O_5_ catalyst for the hydrogenolysis of C–O–C and C–O linkages in various lignins to produce arenes selectively. Additionally, this reaction operates in water as the sole solvent that prevents the formation of ethers from alcohol solvents that are typically used in lignin depolymerization^19^,^20^ and simplify the downstream separation of organic products from the solvent.

### Stability test of the catalyst

The stability of the Ru/Nb_2_O_5_ catalyst for the conversion of birch lignin was tested in four consecutive recycling runs (Supplementary Fig. 3 and Supplementary Table 6). The mass yields of C_7_–C_9_ arenes and C_7_–C_9_ cycloalkanes show little variations between the four runs. Although the used catalyst after four runs show a slight Ru leaching (from 2.0 to 1.6 wt%) and a small decrease of the surface area (from 54 to 41 m^2^ g^−1^) (Supplementary Fig. 8 and Supplementary Table 9), for the fourth run, the mass yield and selectivity of C_7_–C_9_ arenes still reached 20.6 and 70 wt%, respectively, similar to those obtained based on a fresh catalyst. Thus, these results confirmed the high durability of Ru/Nb_2_O_5_ for continuous lignin conversion.

### Model compound studies

**Figure 2:**
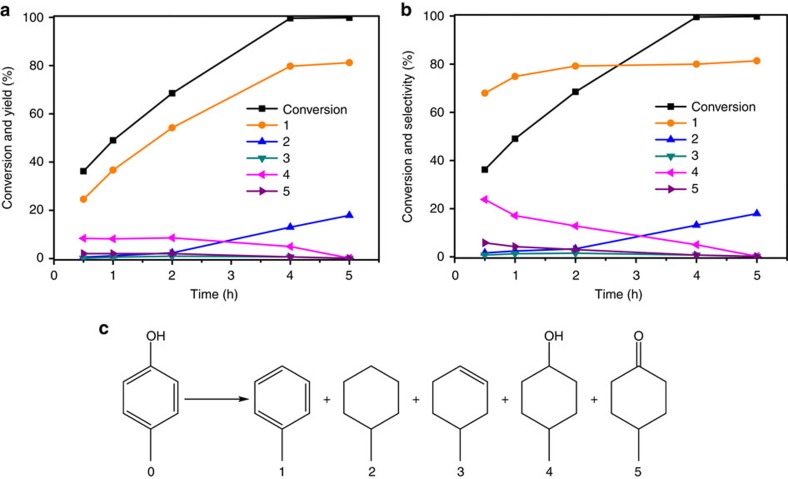
Product distributions for the conversion of 4-methylphenol over Ru/Nb_2_O_5_ versus time. Variation of product yields (**a**) and selectivities (**b**). Reaction conditions: substrate 0.2 g, catalyst 0.4 g, H_2_O 15 ml, 250 °C, H_2_ 0.5 MPa. (**c**) View of the chemical structures for all possible intermediates and products during the hydrodeoxygenation of 4-methylphenol.

The product distribution over the Ru/Nb_2_O_5_ catalyst suggests that the removal of phenolic hydroxyl groups is in direct competition with the hydrogenation of the aromatic phenyl ring. To gain further insights, the conversion of a model compound, 4-methylphenol, over the Ru/Nb_2_O_5_ catalyst was investigated at 250 °C and 0.5 MPa H_2_, and the time profile is shown in Fig. 2.

**Figure 3:**
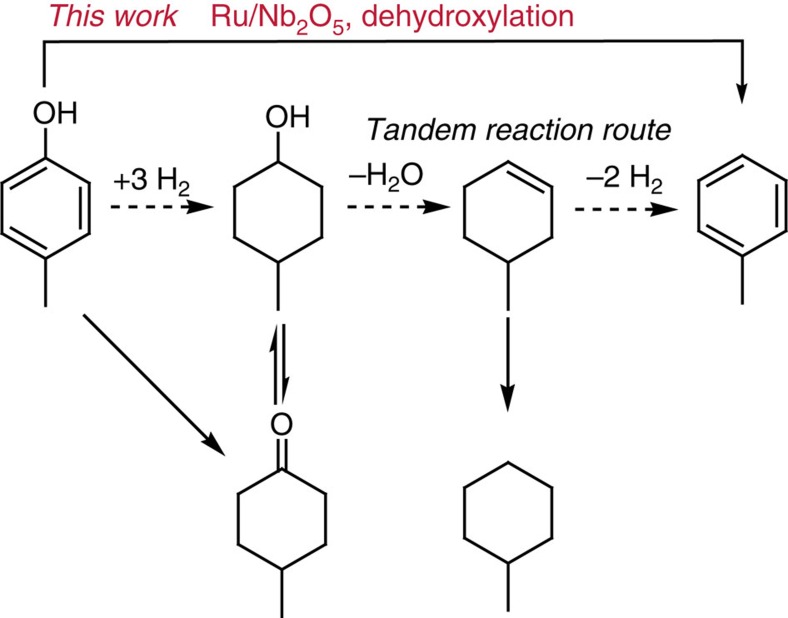
Proposed reaction pathway of the conversion of 4-methylphenol over the Ru/Nb_2_O_5_ catalyst. In this work, toluene is generated through the *C*_aromatic_–OH bond cleavage for direct dehydroxylation of 4-methylphenol over the Ru/Nb_2_O_5_ catalyst and this direct conversion is distinct from the reported tandem reactions route^27^,^32^, where cyclohexene was formed initially, and then dehydrogenated to arenes via hydrogen transfer.

The primary products at low conversion of 36.2% (*t*=0.5 h) consists of toluene (selectivity=68.0%), 4-methylcyclohexanol (selectivity=23.8%) and 4-methylcyclohexanone (selectivity=5.8%), confirming the high arene selectivity within the competitive parallel reactions. As the reaction time prolonged, 4-methylphenol was continuously converted into toluene with a final arene selectivity stabilized at 80%. Upon reaction completion (*t*=5 h), toluene attained a high yield of 81.2%, while the ring-hydrogenated intermediates were completely hydrogenated to methylcyclohexane. It is worth noting that methylcyclohexane solely comes from ring-hydrogenated intermediates (products 3–5 in Fig. 2), rather than from further conversion of toluene because the yield and selectivity of toluene remained constant since *t*=4 h. Interestingly, it is reported that phenol can be converted to arene through a tandem reaction with Ru/sulfate zirconia^32^ or concurrent use of Raney Ni and β-zeolite^27^, where cyclohexene was formed first, and then dehydrogenated to arenes by hydrogen transfer (Fig. 3). In comparison, an equimolar mixture of cyclohexene and 4-methylphenol was used as feedstock over the Ru/Nb_2_O_5_ catalyst to test the dehydrogenation of cyclohexene to yield arenes (Supplementary Fig. 5). The result shows that only 9.8 mol % of benzene was formed and the rest of cyclohexene was hydrogenated to cyclohexane. This result suggests that the main reaction pathway over the Ru/Nb_2_O_5_ catalyst undergoes direct dehydroxylation of 4-methylphenol via a promoted *C*_aromatic_–OH bond cleavage and thus is distinct to the tandem reaction route.

### INS and DFT studies

The selective cleavage of *C*_aromatic_–O bonds in depolymerized lignin components (mostly phenolic compounds) while maintaining the aromatic functionalities of phenyl rings leads to the unusual arene selectivity in this one-pot conversion. Direct observation of the binding dynamics between adsorbed substrates and the catalyst surface is crucial to understand the molecular details of adsorption, activation and hydrodeoxygenation of phenol into arene/alkane (that is, benzene/cyclohexane). INS is well positioned to investigate the dynamics of phenol by exploiting the high neutron scattering cross-section of hydrogen. Additionally, INS can readily access the low energy modes, and is well suited to study the deformational and conformational modes of the –OH group and the C6-rings, respectively. Here we successfully combined INS and DFT calculations to investigate the vibrational and binding properties of phenol molecules on the Ru/Nb_2_O_5_, Ru/ZrO_2_, Ru/Al_2_O_3_ and Ru/TiO_2_ catalysts and the bare Nb_2_O_5_ support to reveal the molecular origins on the hydrodeoxygenation of phenol, representing a unique example of using INS/DFT to study the mechanism for lignin upgrading.

The INS spectrum of the activated Ru/Nb_2_O_5_ catalyst gives a clean background with no prominent features (Supplementary Fig. 14). Upon reduction under H_2_ flow at 150 °C for 3 h, the INS spectrum of the reduced Ru/Nb_2_O_5_ catalyst shows slightly increased intensity overall (Supplementary Fig. 15), indicating the formation of hydrogen-containing species (possibly Nb–OH and/or Ru–H bonds) on the catalyst surface.

**Figure 4:**
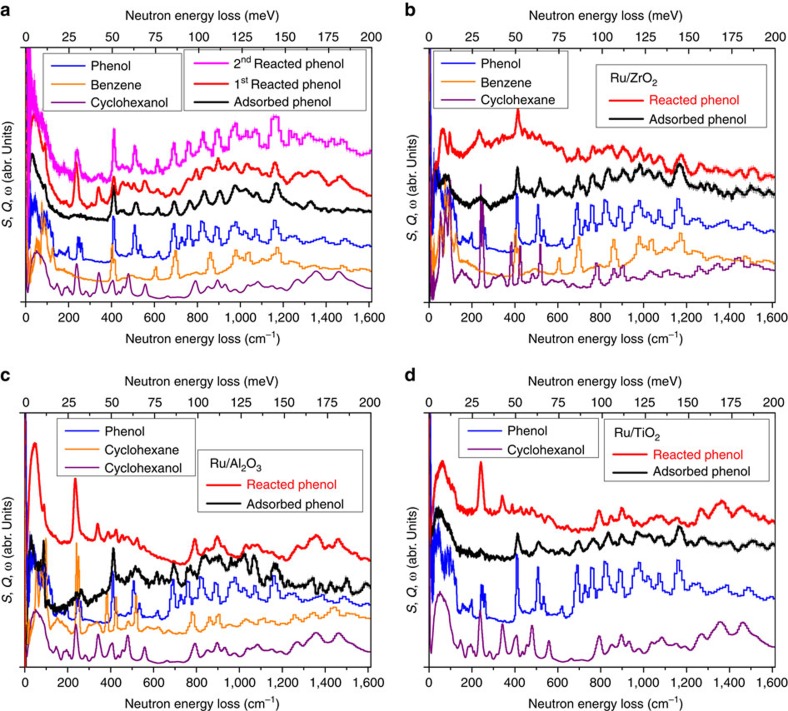
Inelastic neutron scattering (INS) spectra for the catalytic hydrodeoxygenation of phenol. Comparison of the INS spectra for the adsorbed and reacted phenol molecules on Ru/Nb_2_O_5_ (**a**), Ru/ZrO_2_ (**b**), Ru/Al_2_O_3_ (**c**) and Ru/TiO_2_ (**d**) catalysts. All spectra shown here are different spectra; raw data are shown in Supplementary Figs 10–22. INS spectra of the reduced catalysts were used throughout for calculations of the corresponding difference spectra. No abscissa scale factor was used throughout this report for INS calculations. INS spectra for condensed phenol, benzene, cyclohexanol and cyclohexane in solid state at 10 K were included accordingly for a direct comparison of the vibrational modes between the adsorbed/bound molecules and the free intact molecules. Two hydrogenation reactions of adsorbed phenols were conducted on Ru/Nb_2_O_5_ for 5 and 15 min, respectively, given its unique reaction activity. However, a single reaction was conducted on all other catalysts for 5 min only. Where no error bars are visible these are smaller than the symbols used to represent the data points.

Adsorption of phenol was carried out at 140 °C and a notable increase in total intensity and the appearance of a number of new spectral features were clearly observed, consistent with the binding of phenol molecules onto the catalyst surface (Supplementary Figs 16 and 17). Difference INS spectrum showing the signals of adsorbed species was obtained by subtracting the INS spectrum of the reduced catalyst from the phenol adsorbed catalyst (Fig. 4a). A detailed comparison of the difference spectrum and that of condensed phenol in solid at 10 K revealed a number of spectral changes. The low-wavenumber INS peaks (<120 cm^−1^; corresponding to the translational and rotational modes of phenol) shift to lower energy with a continuum profile, suggesting that the adsorbed phenol molecules are disordered over the catalyst surface with a hindered motion as a result of the strong binding to the catalyst. The librational modes of phenol molecules (155–240 cm^−1^) have disappeared completely upon adsorption on the catalyst surface, suggesting that the C6-ring of adsorbed phenol has strongly hindered motion. The modes of C6-ring deformation and out-of-plane wagging of –CH– groups (532 and 724 cm^−1^, respectively) also concurrently disappeared upon binding. The intensity of the –OH bending mode at ∼948 cm^−1^ largely reduced, indicating the deprotonation of the phenol molecules (C_6_H_5_OH→C_6_H_5_O^−^+H^+^) upon adsorption. Similar spectral changes were observed for adsorbed phenol species on the bare Nb_2_O_5_ support (Supplementary Fig. 22), except for a small degree retention of the librational modes of adsorbed phenol molecules at 155–240 cm^−1^, suggesting that the adsorbed phenol molecules are somewhat less immobilized on the bare Nb_2_O_5_ support. The result is reasonable because the Ru loading was only at 2 wt% and thus the majority of adsorption will occur at the Nb_2_O_5_ moiety.

The adsorbed phenol molecules on Ru/Nb_2_O_5_ underwent a first catalytic conversion in H_2_ at 150 °C for 5 min. Comparison of the INS spectra of the first reacted and adsorbed phenol shows appearance of several new features (Fig. 4a). The peaks at 236 and 341 cm^−1^ (corresponding to the C6-ring deformation and ring-conformational modes in cyclohexanol, respectively) indicate the formation of bound cyclohexanoxide on the surface, consistent with the loss of the peak at 189 cm^−1^ (assigned to the –OH wagging mode). The peaks at 398 and 606 cm^−1^ increase in intensity, indicating the formation of weakly adsorbed benzene molecules on the surface as well. This result indicates that the hydrogenation of the phenol C6-ring and the cleavage of C–O bonds are indeed competitive processes at the beginning of the reaction over the Ru/Nb_2_O_5_ catalyst. To probe further into this reaction, a second hydrodeoxygenation reaction was carried out by feeding a H_2_/phenol stream at 150 °C for 15 min aiming for a higher conversion of the adsorbed phenol. The cell outlet was monitored continuously via mass spectrometry that confirmed the presence of the products, benzene and cyclohexane. Comparison of the INS spectra of the first and second reacted phenol on Ru/Nb_2_O_5_ exhibits an interesting observation: the peaks for cyclohexanoxide (for example, 236, 341, 1,350 and 1,450 cm^−1^) are greatly reduced in intensity, indicating that with elongated reaction time, formation of cyclohexanoxide is reduced and the direct cleavage of C–O bonds in phenol is greatly promoted on Ru/Nb_2_O_5_, consistent with the mass spectroscopy (MS) signals. The INS spectrum of reacted phenol molecules on the bare Nb_2_O_5_ catalyst shows very similar features in comparison with the adsorbed state and there is an absence of any hydrogenated product, confirming the critical role of Ru particles in this hydrodeoxygenation reaction, presumably at splitting the molecular H_2_ (Supplementary Fig. 22).

Similar adsorption and reaction of phenol molecules were also carried out on Ru/ZrO_2_, Ru/Al_2_O_3_, and Ru/TiO_2_ catalysts, and *in situ* INS spectra collected (Fig. 4b–d). Adsorption of phenol molecules on these three catalysts is very similar and generally weaker than that over the Ru/Nb_2_O_5_ catalyst as shown by the decreased signal/noise ratio. When compared with the INS spectra of solid phenol, a common feature is that the broad peaks at ∼948, 1,226 and 1,382 cm^−1^ (–OH-related modes) all reduced greatly in intensity, suggesting the deprotonation of adsorbed phenol molecules to form phenoxide on the surface of these three catalysts. Partial retention of the low energy modes (below 120 cm^−1^) suggests a greater motion of adsorbed phenoxide molecules on Ru/Al_2_O_3_ and Ru/TiO_2_ and thus a very weak substrate/catalyst binding interaction. All adsorbed phenoxides underwent one catalytic conversion in H_2_ at 150 °C for 5 min. Interestingly, the reaction intermediates bound/adsorbed on the catalyst surface are found to be different for each catalyst despite a similar adsorption state. The reaction intermediates are found to be cyclohexane/benzene, cyclohexanoxide/cyclohexane and cyclohexanoxide on Ru/ZrO_2_, Ru/Al_2_O_3_ and Ru/TiO_2_, respectively. For the latter two catalysts, cyclohexanoxide is the main product bound on the surface as shown by the marked peak at 236 cm^−1^. In contrast, on Ru/ZrO_2_, the main product is a mixture of alkane/arene. Significantly, the four catalysts studied in the INS experiment show completely different binding/reaction activity, consistent with the observed difference in their catalytic activity on the lignin conversion (Table 1).

**Figure 5:**
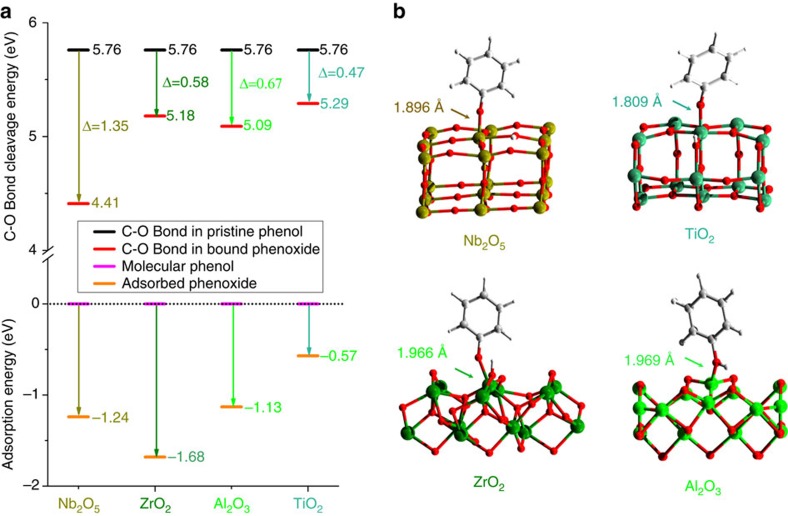
Calculated energies and views of the optimized structural models for phenol binding on catalyst surfaces. (**a**) Calculated energies for phenol adsorption (that is, a combination of deprotonation of *C*_aromatic_–OH to form phenoxide bound to the vacant surface metal sites and the formation of surface –OH groups) and disassociation of the C–O bonds upon adsorption over Nb_2_O_5_, ZrO_2_, Al_2_O_3_ and TiO_2_. (**b**) Views of the corresponding DFT-optimized structural models for the phenoxide bound on Nb_2_O_5_(001), ZrO_2_(010), Al_2_O_3_(110) and TiO_2_(101) surfaces.

DFT calculations were carried out to investigate the adsorption and binding of phenol molecules on the surface of Nb_2_O_5_(001), ZrO_2_(010), Al_2_O_3_(110) and TiO_2_(101) (Fig. 5). In terms of the adsorption of phenol (that is, a combination of deprotonation of *C*_aromatic_–OH to form phenoxide bound to the vacant surface metal sites and the formation of surface –OH groups), ZrO_2_ shows the highest adsorption energy of 1.68 eV. Nb_2_O_5_, Al_2_O_3_ and TiO_2_ show lower adsorption energies of 1.24, 1.13 and 0.57 eV, respectively. Strong adsorption is the first essential step to remove the –OH group in phenol. This DFT calculation is thus in excellent agreement with the INS results, where no cyclohexanoxide was observed on the Ru/ZrO_2_ catalyst owing to its strong and specific adsorption to the –OH groups. Nb_2_O_5_, Al_2_O_3_ and TiO_2_ show weaker adsorption of phenol, and thus cyclohexanoxide was observed on all three catalysts, and as the main product in the latter two cases. In particular, TiO_2_ has the weakest adsorption energy to phenol, leading to an absence of deoxygenated product over the Ru/TiO_2_ catalyst.

To understand the varying selectivities between the competitive processes of *C*_aromatic_–O bond cleavage and C6-ring hydrogenation, we also calculated the disassociation energies for the C–O bond of adsorbed phenoxide on each catalyst. The energy required to cleave C–O bonds in pristine phenol is 5.76 eV, and this is reduced to 4.41, 5.18, 5.09 and 5.29 eV for adsorbed phenoxide on Nb_2_O_5_, ZrO_2_, Al_2_O_3_ and TiO_2_, respectively (Fig. 5). Significantly, Nb_2_O_5_ gives the largest energy reduction (Δ=1.35 eV), suggesting that the cleavage of C–O bond is most greatly promoted here. In contrast, over the other three catalysts, only a moderate energy reduction was seen that, in turn, suggests that the hydrogenation of the phenyl rings can occur more readily than that on Nb_2_O_5_, resulting in the thermodynamically favoured product of alkanes. Significantly, the Nb_2_O_5_ species combines the strong adsorption of phenol and the distinct capability of reducing the disassociation energy for C–O bonds. Once the *C*_aromatic_–O bonds are cleaved and arenes formed, they are generally weakly adsorbed and can desorb from the catalyst surface readily and leave the reaction system rapidly, leading to an overall unusual selectivity towards the production of arenes. ZrO_2_ has solely the strong adsorption capability and is thus more selective to the production of alkanes. Al_2_O_3_ has weaker adsorption than that of ZrO_2_, and leads to the production of both alkanes and oxygenated ring-saturated products. Finally, TiO_2_ displays the weakest adsorption and smallest promotion effect for C–O bond disassociation, and therefore shows mainly the ring-saturated oxygenated products. Overall, this INS/DFT study is in excellent agreement with the catalysis results in Table 1 and has provided molecular origins into these reactions underpinning different product selectivities.

## Discussion

We have presented a catalytic process for one-pot upgrading of raw lignin to hydrocarbons over a Ru/Nb_2_O_5_ catalyst, achieving simultaneously high mass yields and unusual arene selectivities. In this hydrodeoxygenation reaction, phenolic intermediates are formed by the cleavage of *C*_aliphatic_–O ether bonds in lignin, and followed by further hydrogenolysis to arenes. In terms of lignin monomers, the conversion is nearly quantitative. Model compound studies revealed that phenols were converted into aromatic hydrocarbons through the selective cleavage of *C*_aromatic_–OH bonds over the Ru/Nb_2_O_5_ catalyst, and a high temperature favours this process (Supplementary Fig. 9). The uniqueness of niobia support is fully rationalized for this reaction in direct comparison with alumina, titania and zirconia. The superior activity of the Ru/Nb_2_O_5_ catalyst for lignin upgrading originates from a combination of strong adsorption, specific reduction of the disassociation energy for *C*_aromatic_–OH bonds in phenolics upon adsorption and a synergistic effect between Ru and Nb_2_O_5_ species, leading to the high arene selectivity. In comparison with hydropyrolysis, this one-pot approach provides a simpler and more energy-efficient technique for selective conversion of lignin into valuable arene feedstocks.

Although niobium compounds are not widely used in catalysis industries, there are emerging reports describing their catalytic activities^28^,^29^,^30^,^33^. As an early transition metal, niobium has a relatively high abundance of ∼20 p.p.m. in Earth’s crust, similar to cobalt and lithium, but lower than molybdenum and tungsten^34^. The potential of widespread application of niobium-based catalysts in biomass upgrading is strengthened by their high stability; they can be used for several cycles in batch reactors or last for hundreds of hours in fixed-bed reactors^28^,^30^. Finally, recovery of noble metals and niobium from deactivated catalysts can be readily achieved by Aqua and hydrofluoric acid^35^, respectively, further enhancing their promising potential.

## Methods

### Lignin extraction and isolation

Birch lignin was extracted according to a literature procedure^36^. In a 1-litre round-bottom flask with condenser, 40 g of pre-ground birch wood and 400 ml methanol containing 3% hydrogen chloride by weight were combined. The mixture was refluxed for 12 h under stirring, and cooled to room temperature. Residue was removed by suction filtration and washed with additional small portions of methanol. The filtrate was concentrated to <200 ml by rotary evaporation and then poured into 1-litre of ice-cold water with vigorous stirring, causing a light brown solid to precipitate. This lignin was collected by filtration, washed with a small portion of water and dried under vacuum. The yield of dried, crude birch lignin was 4.97 g (12.4 wt%). Beech lignin and pine lignin were extracted in the same way, and the yield was 4.94 g (12.3 wt%) and 3.68 g (9.2 wt%), respectively. The Gel Permeation Chromatography (Waters 1515) was performed to analyse the molecular weights of the extracted lignin. Before analysis, lignin was dissolved in tetrahydrofuran. A calibration curve was obtained using monodisperse polystyrene standards.

### Catalyst preparation

Nb_2_O_5_ was synthesized according to published procedures^28^. The Ru-based catalysts were prepared by the incipient wetness impregnation method with appropriate amounts of aqueous solution of RuCl_3_. The obtained sample was dried at 100 °C for 12 h and then reduced in a 10% H_2_/Ar flow at 400 °C for 3 h. The metal loading in each catalyst was at 2 wt%. Other supports (ZrO_2_, Al_2_O_3_, TiO_2_, H-ZSM-5 and activated carbon) loaded Ru catalysts were also prepared with incipient wetness impregnation method.

### Catalytic test and product analysis

The detailed reaction conditions are described in the figure captions and table footnotes. In a typical reaction, catalyst (0.2 g) and lignin (0.1 g) were loaded into a stainless-steel autoclave reactor (Anhui Kemi Machinery Technology Co., Ltd) with water (15 ml) as the solvent. After the reactor was purged with H_2_ three times and charged with 0.7 MPa H_2_, the reaction was conducted at 250 °C with a magnetic stirring speed of 600 r.p.m. After the reaction, the reactor was quenched to ambient temperature in an ice-water bath, and the organic products were extracted using ethyl acetate and analysed by gas chromatography (GC) and GC–MS on an Agilent 7890B gas chromatograph with flame ionization detector and an Agilent 7890A GC-MS instrument, both equipped with HP-5 capillary columns (30 m × 250 μm). Tridecane was used as an internal standard for the quantification of the liquid products. For the stability test, after each reaction, the used catalyst was isolated, briefly washed with ethanol to remove the unreacted lignin feedstock and dried at 80 °C in air before the next run.

### *In situ* INS

INS spectra were recorded on the VISION spectrometer at Spallation Neutron Source, Oak Ridge National Laboratory (USA), as well as the TOSCA spectrometer at the ISIS Facility at the STFC Rutherford Appleton Laboratory (UK). Both VISION and TOSCA are indirect geometry crystal analyser instruments that provide a wide dynamic range with high resolution. All the INS spectra for the catalysis system were collected after the sample was cooled and stabilized at temperatures below 30 K.

The adsorption/hydrogenation experiments were conducted under static and flow conditions at beamlines VISION and TOSCA, respectively. Results obtained from both conditions are consistent. In a typical experiment, ∼15–22 g catalyst was loaded into a flow-type stainless steel cell that can also be used as a static cell with all valves closed. Except for the bare Nb_2_O_5_ support, all catalysts used here have a 2 wt% loading of Ru. By heating at 300 °C under He flow for 3 h, the trace amount of water that was adsorbed on the catalyst surface was removed. The activated catalyst was then reduced by heating under a H_2_ flow at 150 °C for 3 h. To study the reaction mechanism, phenol was used as a model compound and dosed into the sample cell at 140 °C for 3 h. The amount of phenol to be dosed into the catalysis cell was calculated based on the surface area and mass of the catalyst loaded in the cell to achieve a monolayer adsorption for each catalyst to enable a direct comparison between them. The INS spectrum of the phenol adsorbed catalyst was collected after cooling to below 30 K. Before the data collection, the cell was flushed by dry He to remove weakly bound phenol molecules. After the data collection, H_2_ was introduced to the cell for 5 min at 150 °C for the catalytic reaction to occur. In the case of Ru/Nb_2_O_5_, a second catalytic reaction was conducted under a H_2_/phenol flow for 15 min at 150 °C to enrich the products on the catalyst surface. The exhaust gas was monitored via mass spectrometry.

### Condensed substrate molecules in solid

INS spectra of pure solid compounds for both starting material and reaction products were collected at 10 K. The amount of each sample in the neutron beam is: phenol: 2.4 g; benzene: 8.1 g; cyclohexane: 7.1 g; and cyclohexanol: 8.0 g.

### Data availability

The data supporting the findings of this study are available within the article, or available from the authors on reasonable request.

## Additional information

**How to cite this article:** Shao, Y. *et al*. Selective production of arenes via direct lignin upgrading over a niobium-based catalyst. *Nat. Commun.* **8,** 16104 doi: 10.1038/ncomms16104 (2017).

**Publisher’s note**: Springer Nature remains neutral with regard to jurisdictional claims in published maps and institutional affiliations.

## Supplementary Material

Supplementary Information Supplementary Figures and Supplementary Methods

## Figures and Tables

**Figure 1:**
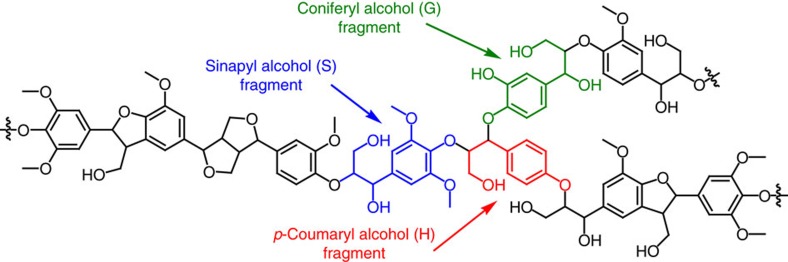
Schematic representation of a typical lignin structure. Lignin is a complex aromatic biopolymer that is constructed from monomers of coumaryl, coniferyl and sinapyl alcohols via intermolecular C–O and C–C bonds in a random order.

**Table 1:**
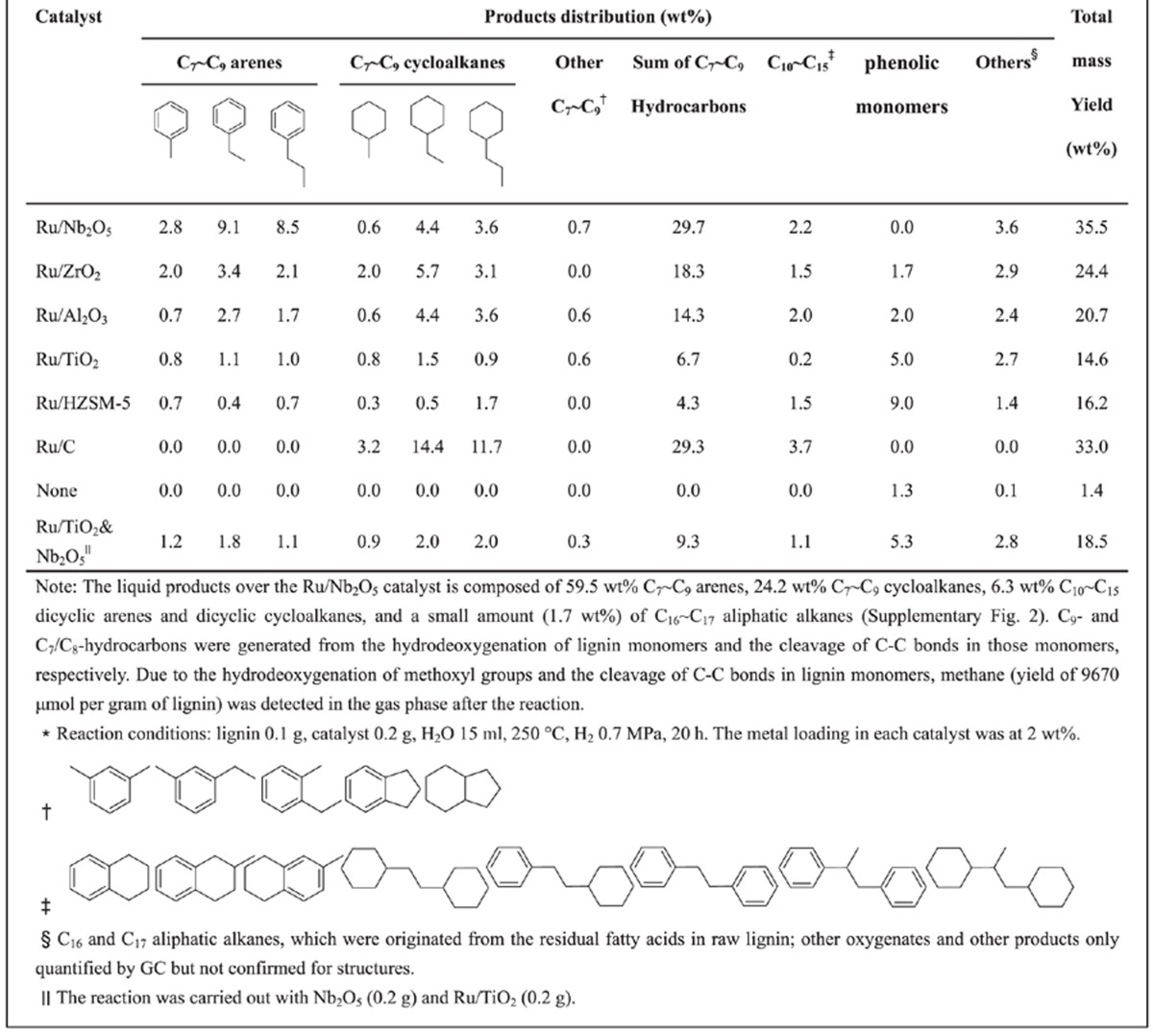
Summary of product yields from direct hydrodeoxygenation of birch lignin*.
