# Supplementary material for: Selective production of arenes via direct lignin upgrading over a niobium-based catalyst
Source: Nat Commun. 2017 Jul 24;8:16104. doi: 10.1038/ncomms16104 (PMC5527281; doi:10.1038/ncomms16104)
Supplement: Supplementary Information — Supplementary Figures and Supplementary Methods [file ncomms16104-s1.pdf]

Supplementary Figures

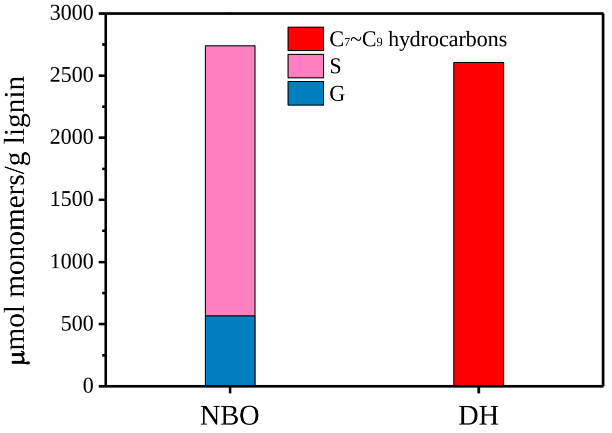

Supplementary Figure 1. Birch lignin monomer analysis. “NBO” refers to the nitrobenzene oxidation method; “DH” refers to direct hydrogenolysis over Ru/Nb_2O_5; “S” refers to syringy units; “G” refers to guaiacyl units.

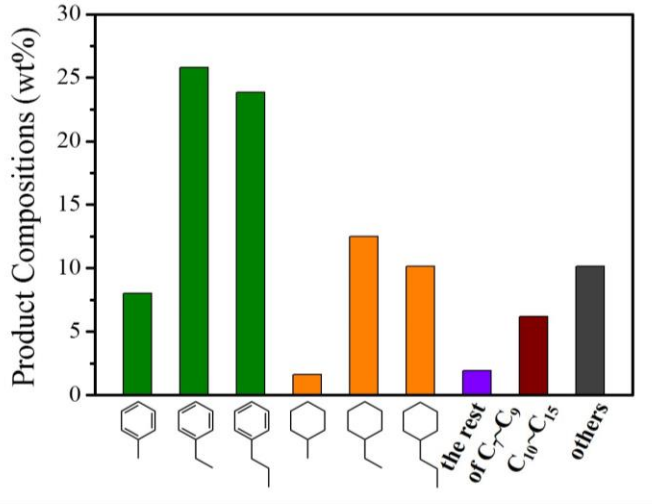

Supplementary Figure 2. Product distribution in the liquid phase from birch lignin conversion over the Ru/Nb_2O_5 catalyst. Reaction conditions: lignin 0.1 g, Ru/Nb_2O_5 0.2 g, H_2O 15 mL, 250 °C, H_2 0.7 MPa, 20h.

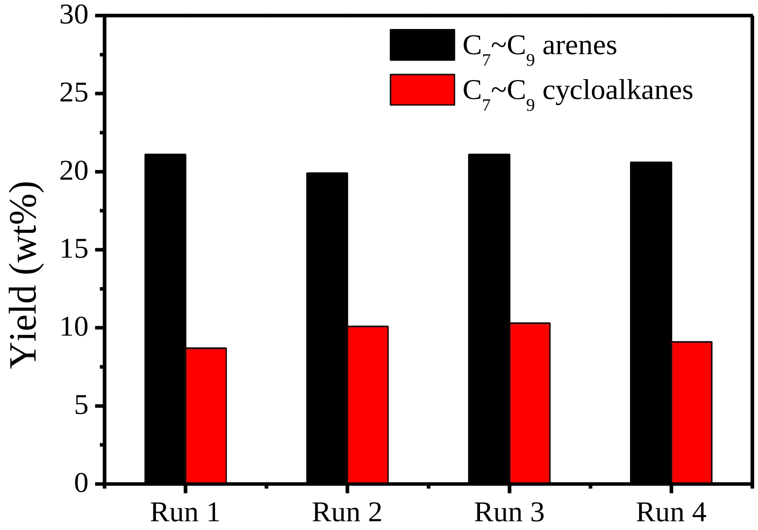

Supplementary Figure 3. Yield and selectivity of the stability test of lignin conversion over the Ru/Nb_2O_5 catalyst. Reaction conditions: lignin 0.1 g, Ru/Nb_2O_5 0.2 g, H_2O 15 mL, 250 °C, H_2 0.7 MPa, 20h.

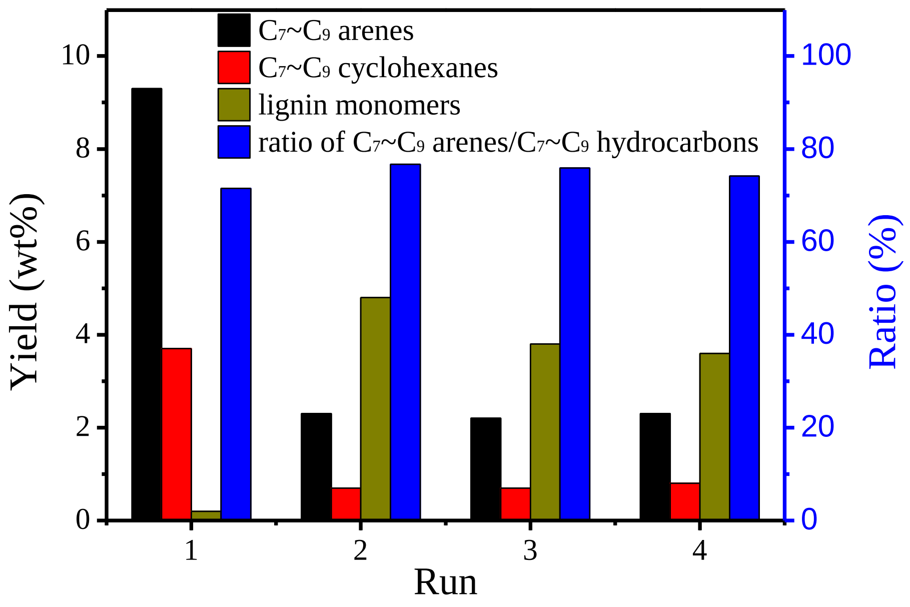

Supplementary Figure 4. Recycling tests of lignin conversion over Ru/Nb_2O_5 catalyst with substrate (0.1 g)/catalyst (0.1 g) ratio. Reaction conditions: lignin 0.1 g, Ru/Nb_2O_5 0.1 g, H_2O 15 mL, 250 °C, H_2 0.7 MPa, 20h.

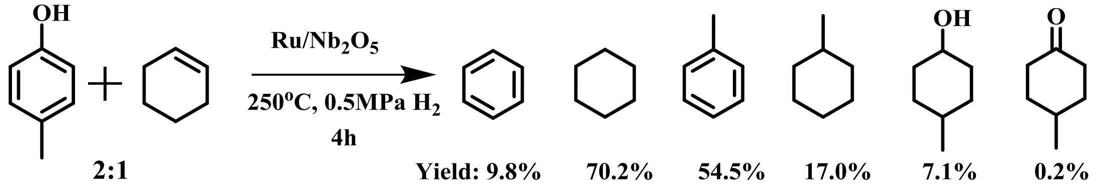

Supplementary Figure 5. Product distribution for the conversion of the equimolar mixture of cyclohexene and 4-methylphenol over the Ru/Nb_2O_5 catalyst. Reaction conditions: 4-methylphenol 0.2 g, cyclohexene 0.076g, 2%Ru/Nb_2O_5 0.4 g, H_2O 15 mL, 250 °C, H_2 0.5 MPa, 4h.

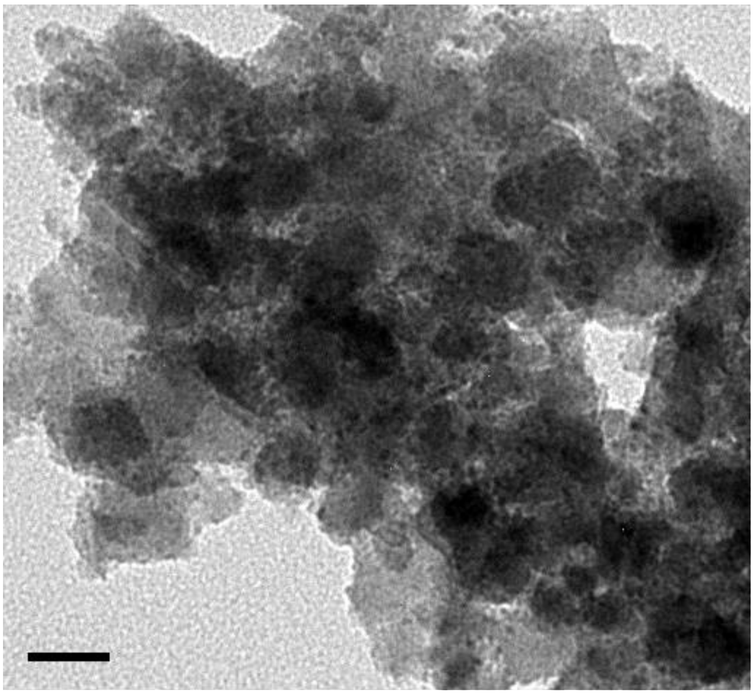

Supplementary Figure 6. TEM image of a fresh 2wt%Ru/Nb_2O_5 catalyst (scale bar 20 nm). The Ru particles are around 1-3 nm and homogeneously distributed on the Nb_2O_5 support.

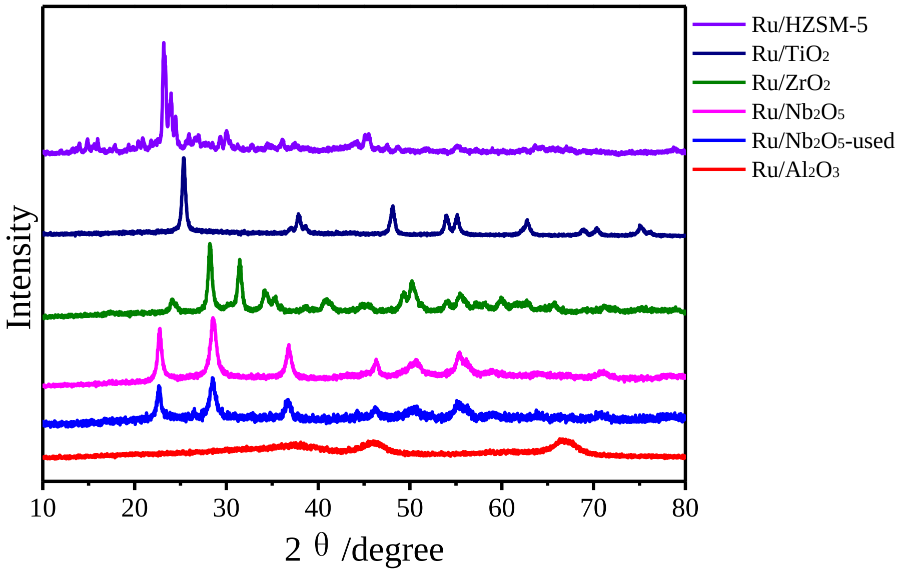

Supplementary Figure 7. XRD patterns of all supported Ru catalysts used in this study.

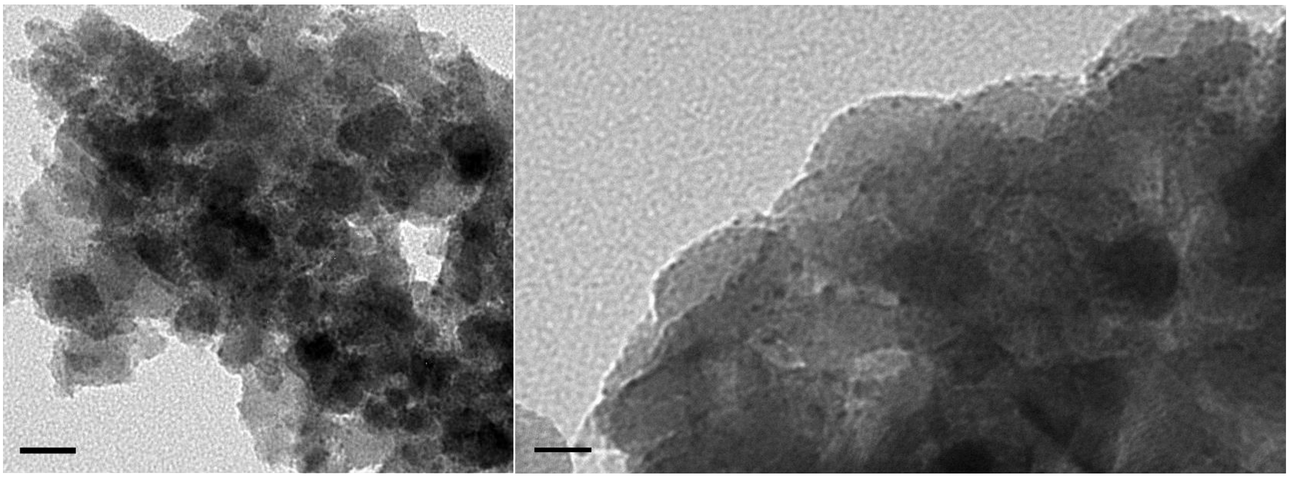

a b

Supplementary Figure 8. TEM images of a fresh Ru/Nb_2O_5 catalyst (a, scale bar 20 nm) and a used Ru/Nb_2O_5 catalyst (b, scale bar 10 nm) after four recycling reactions of lignin conversion. The diameters of Ru particles for both fresh and used catalysts are mainly distributed between 1 and 3 nm. No clear sintering of Ru particles was found.

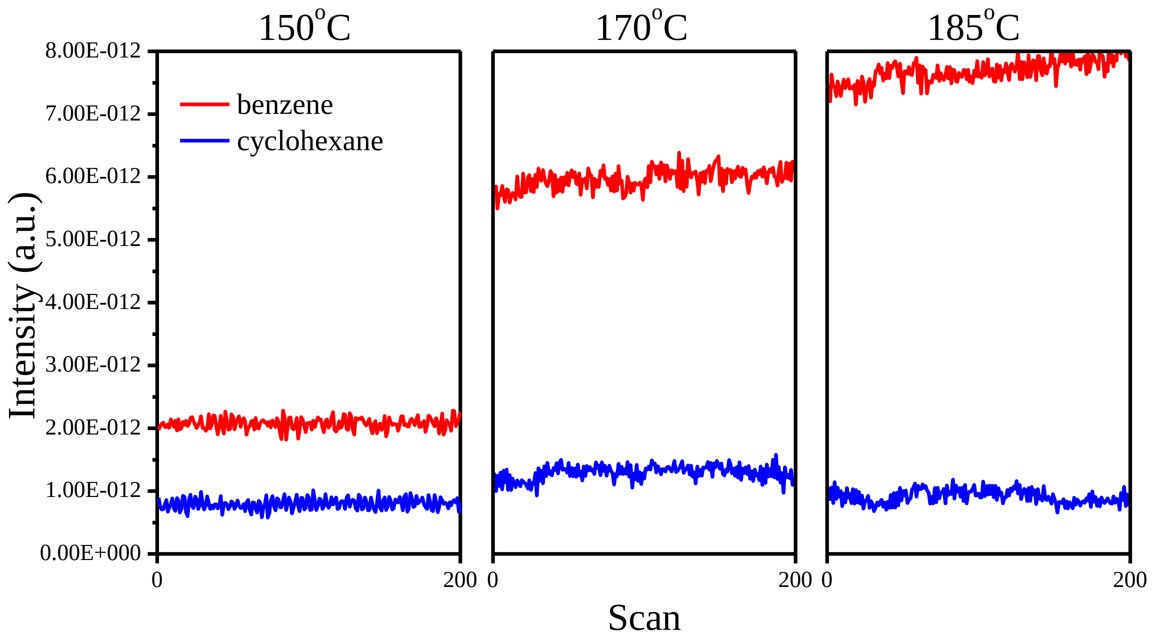

Supplementary Figure 9. The MS signals of benzene (*m/z* = 78) and cyclohexane (*m/z* = 84) in the *in situ* surface reaction over the Ru/Nb_2O_5catalyst at different temperatures.

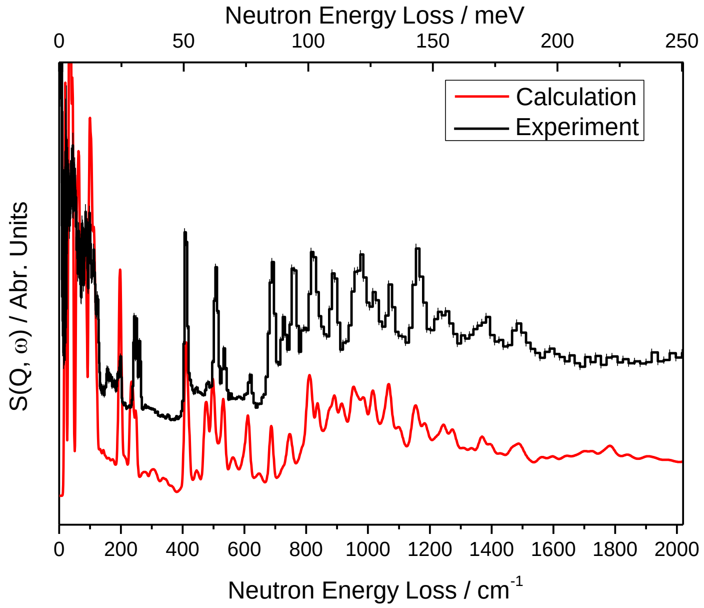

Supplementary Figure 10. Comparison of calculated and experimental INS spectra of condensed phenol in solid at 10K.

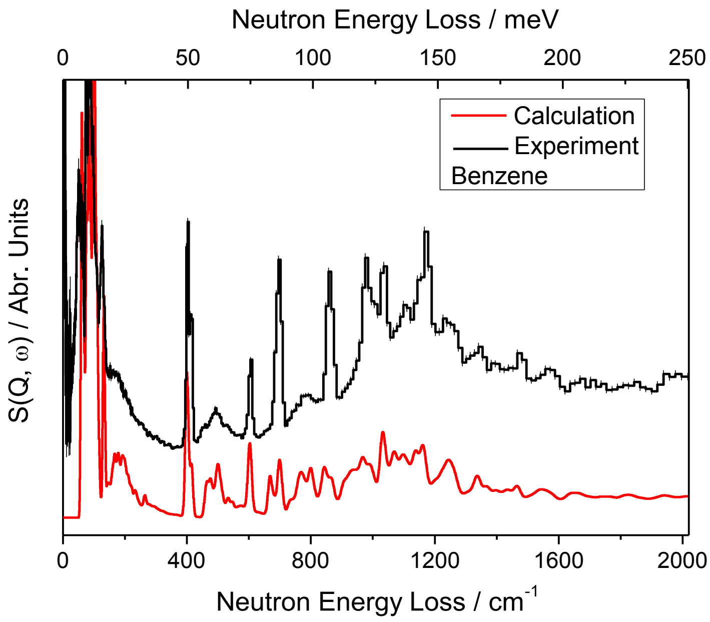

Supplementary Figure 11. Comparison of calculated and experimental INS spectra of condensed benzene in solid at 10K.

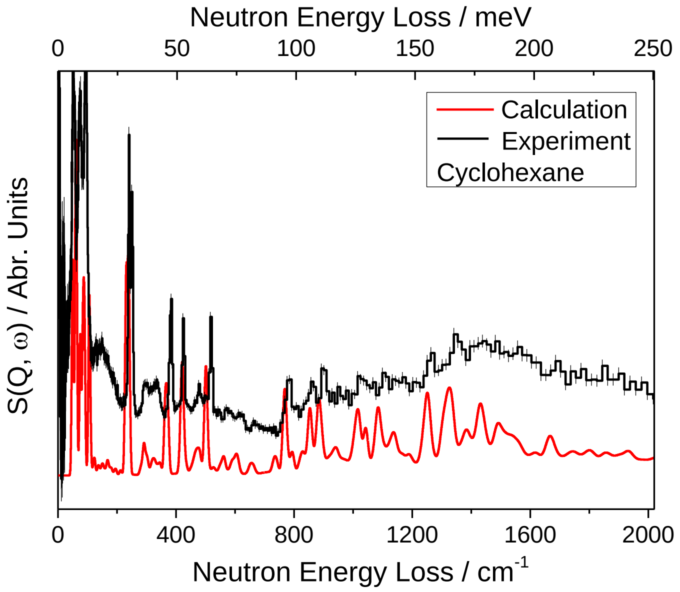

Supplementary Figure 12. Comparison of calculated and experimental INS spectra of condensed cyclohexane in solid at 10K.

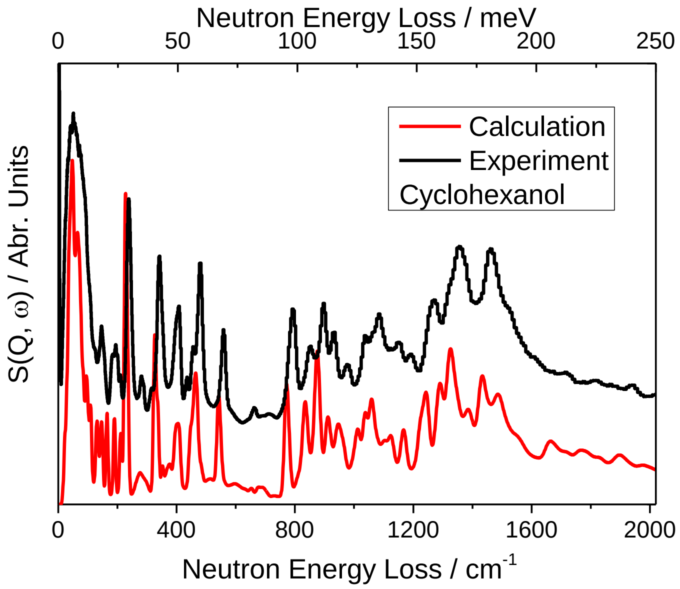

Supplementary Figure 13. Comparison of calculated and experimental INS spectra of condensed cyclohexanol in solid at 10K.

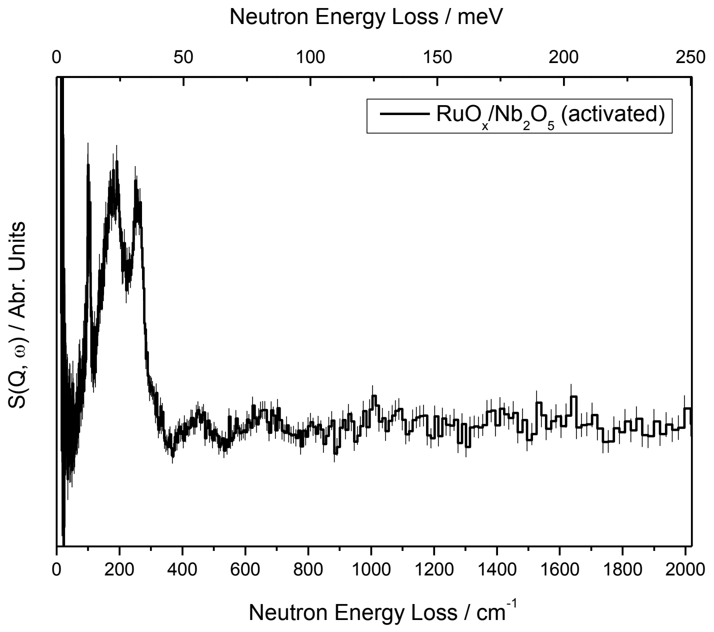

Supplementary Figure 14. INS spectrum of the activated RuO_x/Nb_2O_5 catalyst. The features below 300 cm^{-1} and at approximately 448 and 655 cm^{-1} are contributed by the stainless steel catalysis cell.

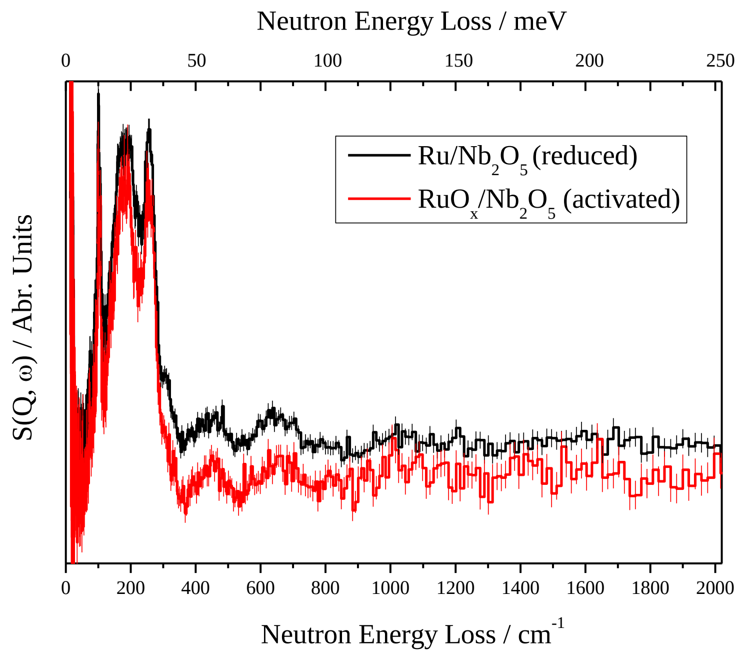

Supplementary Figure 15. Comparison of INS spectra of the activated and reduce Ru/Nb_2O_5 catalysts.

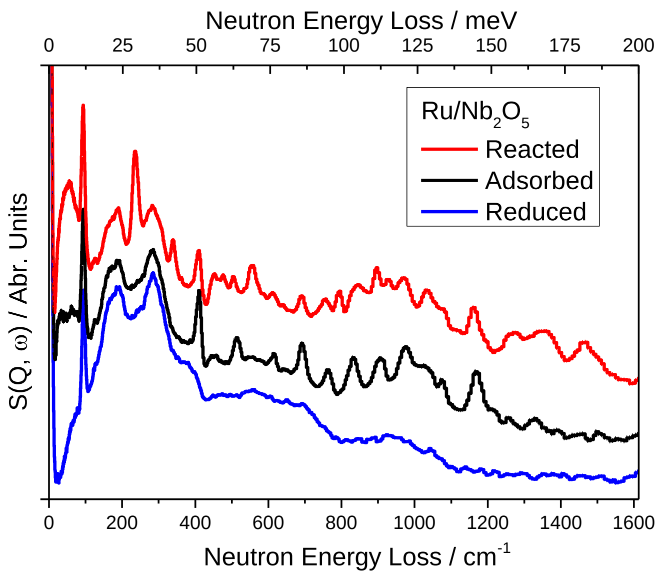

Supplementary Figure 16. Comparison of the INS spectra for the bare, phenol-adsorbed and reacted catalyst Ru/Nb_2O_5.

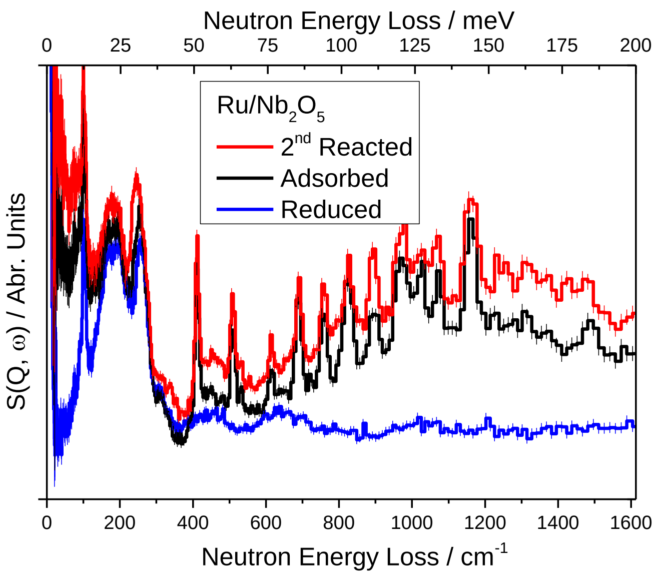

Supplementary Figure 17. Comparison of the INS spectra for the bare, phenol-adsorbed and reacted catalyst Ru/Nb_2O_5 for a second reaction.

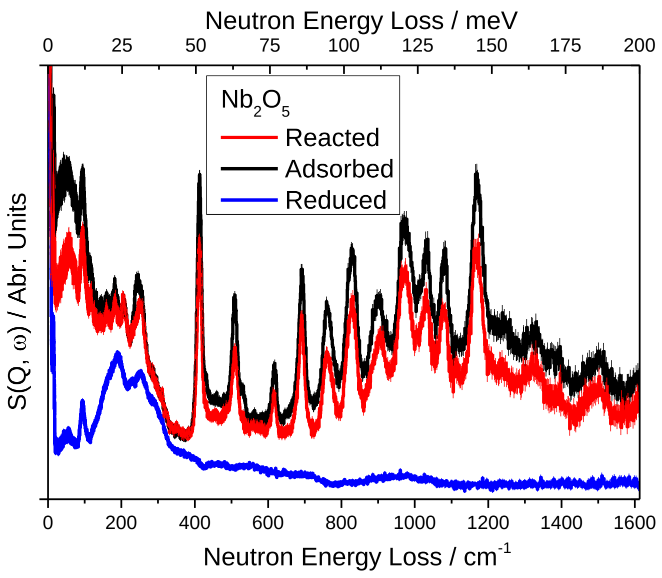

Supplementary Figure 18. Comparison of the INS spectra for the bare, phenol-adsorbed and reacted catalyst Nb_2O_5.

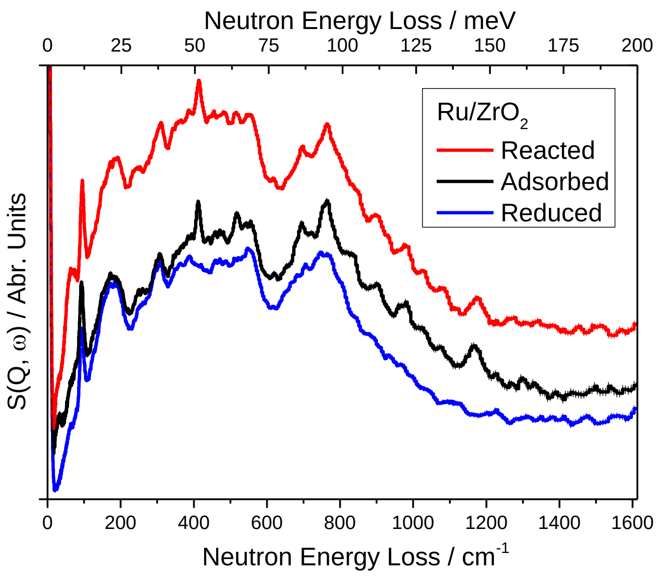

Supplementary Figure 19. Comparison of the INS spectra for the bare, phenol-adsorbed and reacted catalyst Ru/ZrO_2.

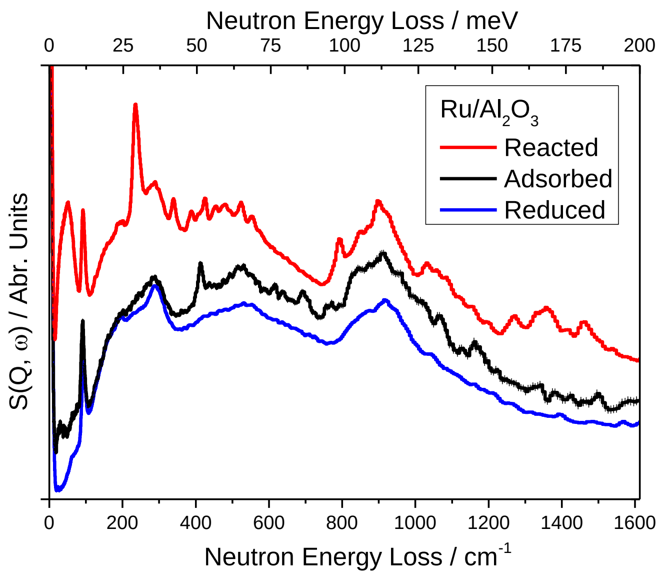

Supplementary Figure 20. Comparison of the INS spectra for the bare, phenol-adsorbed and reacted catalyst Ru/Al_2O_3.

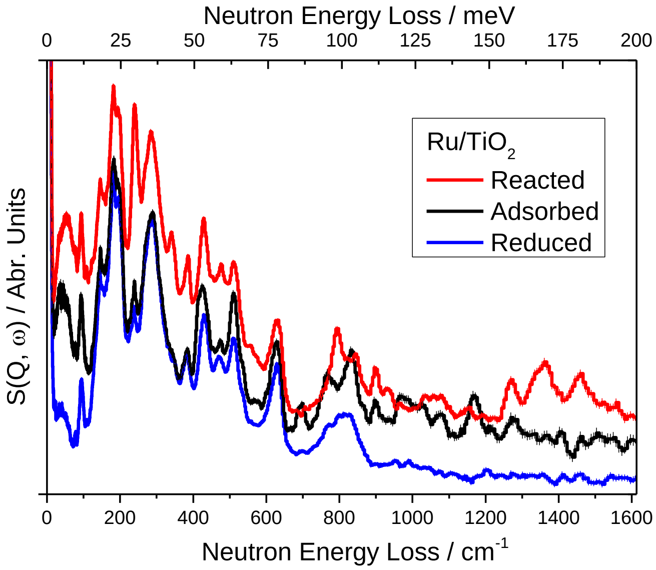

Supplementary Figure 21. Comparison of the INS spectra for the bare, phenol-adsorbed and reacted catalyst Ru/TiO_2.

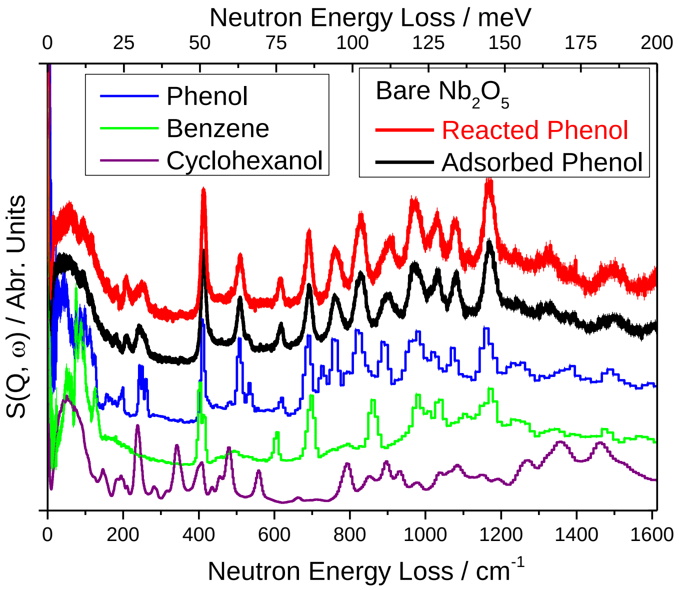

Supplementary Figure 22. Comparison of the INS spectra for the adsorption and hydrodeoxygenation reaction of phenol on the bare Nb_2O_5 support, confirming the absence of formation of the hydrogenated product.

Supplementary Tables

Supplementary Table 1. The GC-MS identified liquid products, their retention time and yields after birch lignin conversion for 20h over Ru/Nb_2O_5. The GC data was obtained from a FID detector. In addition to these products shown below, there are other 1.87 wt% (yield) products quantified by GC but not confirmed for their structures, because their peak areas on GC-MS are too small to be identified with structure by MS. Reaction conditions: lignin 0.1 g, Ru/Nb_2O_5 0.2 g, H_2O 15 mL, 250 °C, H_2 0.7 MPa, 20h.

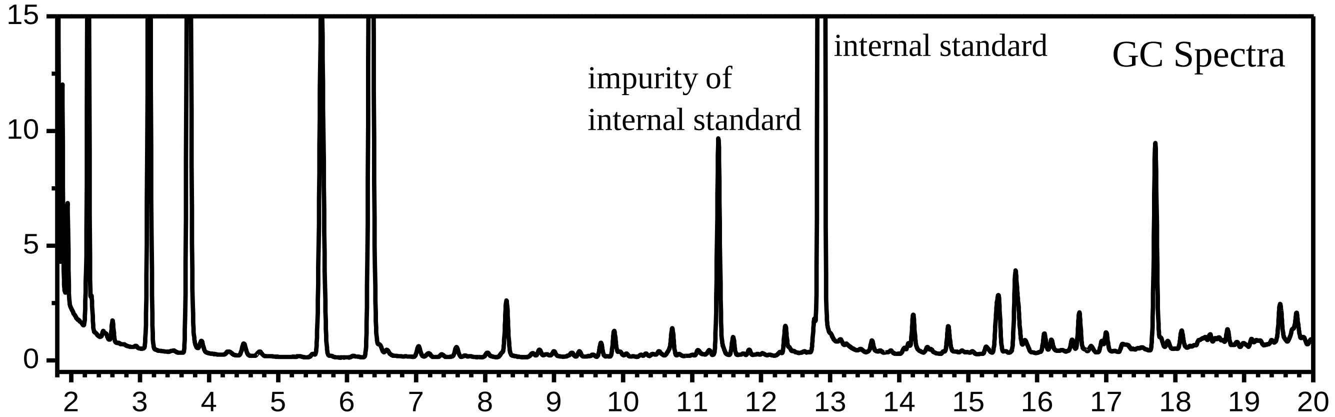

No.	Ret. time (min)	Compound	Yield (wt%)	No.	Ret. time (min)	Compound	Yield (wt%)
1	1.872	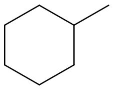	0.6	11	10.711	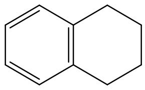	0.2
2	2.247	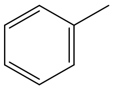	2.8	12	11.594	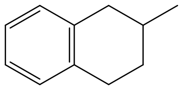	0.1
3	3.132	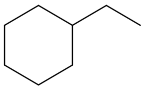	4.4	13	12.352	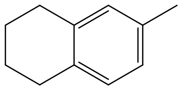	0.2
4	3.704	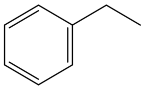	9.1	14	15.436	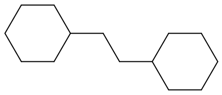	0.6
5	4.504	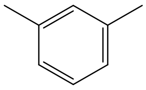	0.1	15	15.687	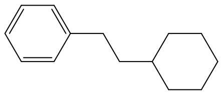	0.8
6	5.628	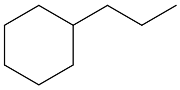	3.6	16	15.823	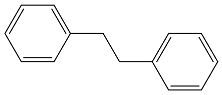	0.1
7	6.344	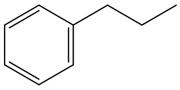	8.5	17	16.104	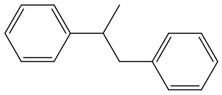	0.1
8	7.036	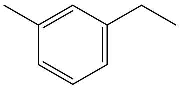	0.1	18	16.206	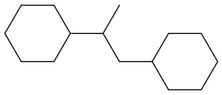	0.1

9	7.585	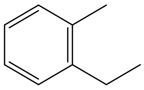	0.1	19	16.611	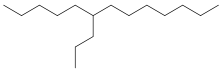 *i*-C_{16}	0.2
10	8.310	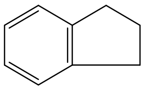	0.4	20	17.712	*n*-C_{17}	1.5

Supplementary Table 2. The GC-MS identified liquid products, their retention time and yields after birch lignin conversion for 10h over Ru/Nb_2O_5. In addition to these products shown below, there are other small amounts of products with unconfirmed structures, because their peak areas on GC-MS are too small to be identified with structure by MS. Reaction conditions: lignin 0.1 g, Ru/Nb_2O_5 0.2 g, H_2O 15 mL, 250 °C, H_2 0.7 MPa, 10h.

No.	Ret. time (min)	Compound	Yield (wt%)	No.	Ret. time (min)	Compound	Yield (wt%)
1	1.872	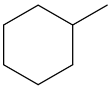	0.1	6	6.344	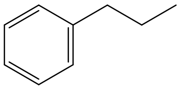	2.3
2	2.247	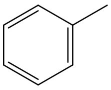	0.9	7	12.673	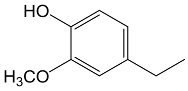	1.2
3	3.132	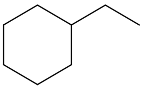	2.1	8	13.837	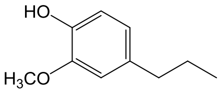	0.9
4	3.704	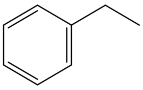	2.7	9	15.811	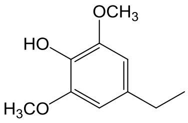	2.7
5	5.628	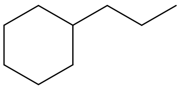	1.6	10	16.775	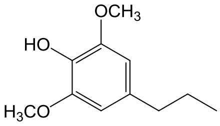	2.8

Supplementary Table 3. Main product yields from direct hydrodeoxygenation of birch lignin over different catalysts.

Catalyst	Products distribution (wt%)
Ru/ZrO_2	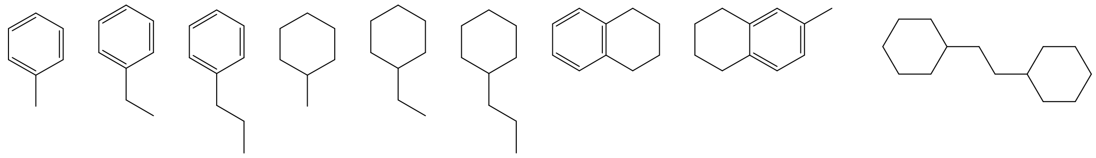<br>2.0 3.4 2.1 2.0 5.7 3.1 0.1 0.2 0.8
	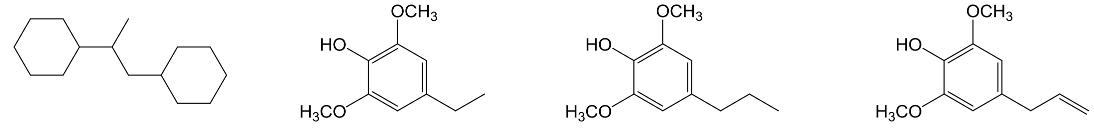<br>0.4 0.6 0.2 0.9
	n-C_{17}: 1.7 Others: 1.2
Ru/Al_2O_3	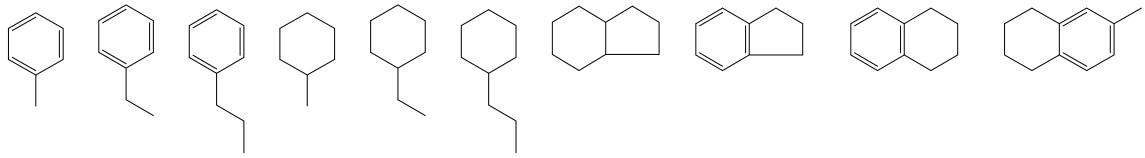<br>0.7 2.7 1.7 0.6 4.4 3.6 0.2 0.4 0.2 0.4
	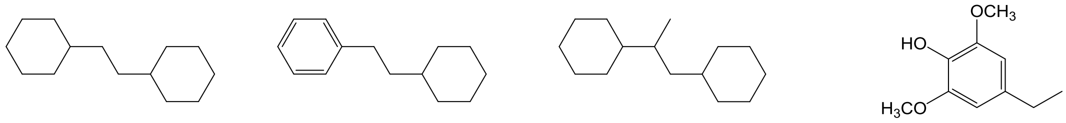<br>0.9 0.3 0.2 1.5
	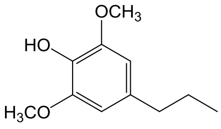 0.5 Others: 2.4
Ru/TiO_2	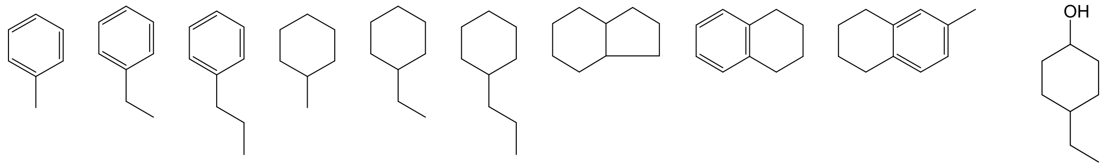<br>0.8 1.1 1.0 0.8 1.5 0.9 0.4 0.2 0.2 1.2
	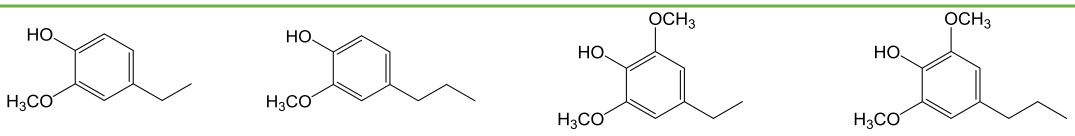<br>0.3 0.6 2.2 1.9

	Others									
	1.5									
Ru/ H-ZSM-5	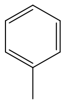	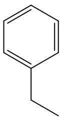	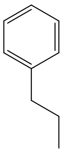	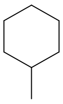	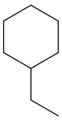	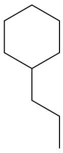	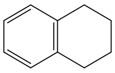	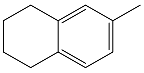	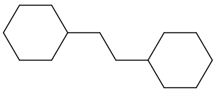	
	0.7	0.4	0.7	0.3	0.5	1.7	0.1	0.4	0.4	
	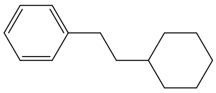	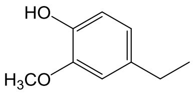	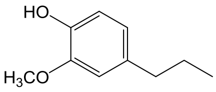	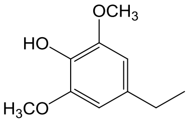						
	0.6	0.4	1.2	2.0						
	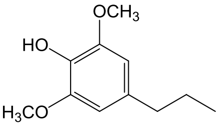	Others								
	5.4	1.4								
Ru/C	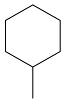	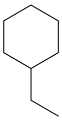	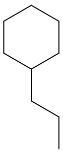	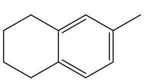	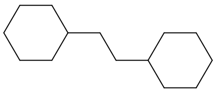	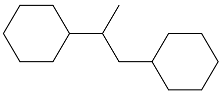				
	3.2	14.4	11.7	0.2	2.3	1.2				
None	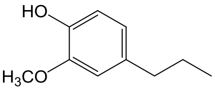	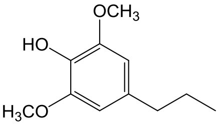	Others							
	0.2	1.1	0.1							
Ru/TiO_2& Nb_2O_5	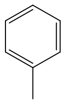	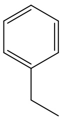	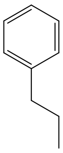	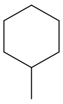	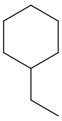	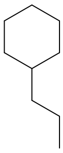	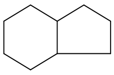	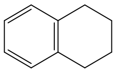	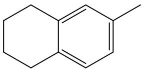	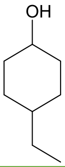
	1.2	1.8	1.1	0.9	2.0	2.0	0.3	0.2	0.9	1.2
	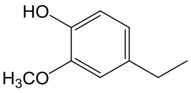	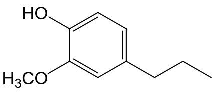	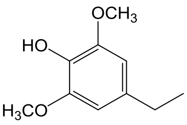	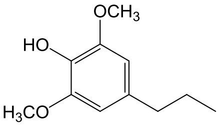						
	0.4	0.7	2.1	2.1						
	Others									
	1.6									

[a] Reaction conditions: lignin 0.1 g, catalyst 0.2 g, H_2O 15 mL, 250 °C, H_2 0.7 MPa, 20h.

Supplementary Table 4. Main product yields from direct hydrodeoxygenation of birchlignin over Ru/Nb_2O_5 with different substrate/catalyst ratios. Reaction conditions: lignin 0.1 g, H_2O 15 mL, 250 °C, H_2 0.7 MPa, 20h.

Amount of catalyst (g)	Products distribution (wt%)										Ratio of C_7~C_9 arenes/C_7~C_9 Hydrocarbons (wt%)
	C_7~C_9 arenes			C_7~C_9 cycloalkanes			Other C_7~C_9[a]	Sum of C_7~C_9 Hydrocarbons	C_{10}~C_{15}[b]	phenolic monomers	
	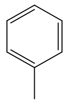	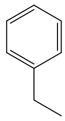	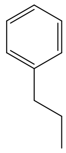	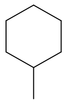	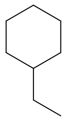	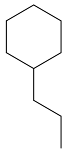					
0.2	2.8	9.1	8.5	0.6	4.4	3.6	0.7	29.7	2.2	0.0	71.0
0.1	1.4	4.4	3.5	0.4	2.0	1.3	0.2	13.2	1.7	0.2	71.9
0.05	0.6	1.2	1.0	0.3	0.6	0.6	0.0	4.3	0.0	8.9	65.1

[a] 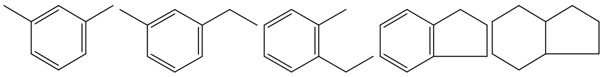

[b] 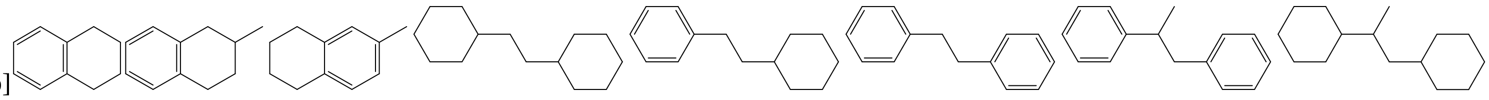

Supplementary Table 5. Main product yields from direct hydrodeoxygenation of various lignins over Ru/Nb_2O_5catalyst.[a]

Substrate	Products distribution (wt%)									Total weight Yield (wt%)
	C_7~C_9 arenes			C_7~C_9 cycloalkanes			Other C_7~C_9	C_{10}~C_{15}	Others	
	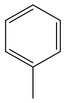	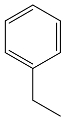	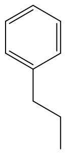	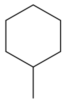	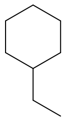	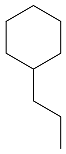				
birch	2.8	9.1	8.5	0.6	4.4	3.6	0.7	2.2	3.6	35.5
beech	3.5	6.7	8.3	1.3	3.0	3.1	1.1	2.1	1.1	30.2
pine	3.7	5.0	5.0	1.2	2.6	1.8	0.6	4.9	1.9	26.7

[a] Reaction conditions: lignin 0.1 g, 2%Ru/Nb_2O_5 0.2 g, H_2O 15 mL, 250 °C, H_2 0.7 MPa, 20h.

Supplementary Table 6. Main product yields from recycling tests of birch lignin conversion over Ru/Nb_2O_5 catalyst.[a]

Run	Products distribution (wt%)								Percent of C_7~C_9 arenes to the total C_7~C_9 products (wt%)
	C_7~C_9 arenes				C_7~C_9 cycloalkanes				
	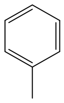	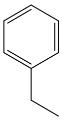	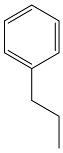	Total	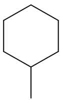	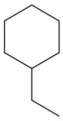	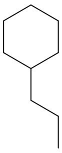	Total	
1	2.8	9.1	8.5	21.1	0.6	4.4	3.6	8.6	71.0
2	3.7	8.8	7.4	19.9	1.2	5.6	3.8	10.1	66.3
3	3.0	10.9	7.2	21.1	1.0	6.1	3.2	10.3	67.2
4	3.4	9.6	7.1	20.6	1.3	4.5	3.2	9.1	69.4
1[b]	3.7	8.7	7.8	20.2	1.1	5.1	3.7	9.9	67.1
1[b]	3.7	9.0	7.4	20.1	1.8	5.2	3.5	10.5	65.7

[a] Reaction conditions: lignin 0.1 g, 2%Ru/Nb_2O_5 0.2 g, H_2O 15 mL, 250 °C, H_2 0.7 MPa, 20h.

[b] Further test of birch lignin conversion was conducted three times each with a fresh Ru/Nb_2O_5 catalyst, showing a small fluctuation between different runs.

**Supplementary Table 7. GPC analysis of isolated lignin from different woods.**			
Sample	M_w (g mol^{-1})	M_n (g mol^{-1})	M_w/M_n
Birch lignin	3214	1700	1.89
Beech lignin	2865	1530	1.87
Pine lignin	2644	1460	1.81

Supplementary Table 8. BET surface area of different materials.

Sample	S_{BET} /m^2g^{-1}
Nb_2O_5	70.0
HZSM-5	298.0
TiO_2	29.0
ZrO_2	2.0
Al_2O_3	197.0
Activated carbon	689.2

Supplementary Table 9. Characterisation of fresh and used Ru/Nb_2O_5 catalysts.		
Sample	Ru content by ICP/wt%	S_{BET} /m^2g^{-1}
2%Ru/Nb_2O_5-fresh	2.0	54
2%Ru/Nb_2O_5-used[a]	1.6	41

[a] Used catalyst after four recycling measurements of lignin conversion.

Supplementary Table 10. Comparison of MS signals of benzene (*m/z* = 78) and cyclohexane (*m/z* = 84) in *in situ* surface reaction at different temperatures.

Catalyst	T (°C)	Intensity (10^{-12} a.u.)		benzene/cyclohexane ratio
		Benzene (*m/z*=78)	Cyclohexane (*m/z*=84)	
Ru/Nb_2O_5	150	2.0	0.8	2.5
	170	5.9	1.3	4.5
	185	7.5	0.8	9.4

Supplementary Note 1: Analysis of lignin monomers by alkaline nitrobenzene oxidation method (NBO).

NBO was performed following a reported literature procedure[5,6]. In a typical reaction, extracted birch lignin (40 mg) was mixed with nitrobenzene (0.4 mL) and 2 M NaOH (7 mL) and reacted at 170 °C for 2 h in an oil bath. Afterwards, the reactor was cooled in ice-water and 1 mL of freshly prepared ethyl vanillin (3-ethoxy-4-hydroxybenzaldehyde, **EV**) (5 μmol/mL) in 0.1 M NaOH solution was added to the reaction mixture as an internal standard. The mixture was transferred to a 100-mL separation funnel and washed three times with 15 mL of dichloromethane. The remaining aqueous layer was acidified with 2 M HCl, until the pH was below 3.0 and extracted twice with 20 mL of dichloromethane and 20 mL of diethyl ether. The combined organic layer was washed with deionised water (20 mL) and dried over Na_2SO_4. After filtration, the filtrate was collected in a 100-mL pear-shaped flask and dried under reduced pressure. For the TMS (trimethylsilyl) derivatisation step, NBO-products were washed with pyridine (3 × 200 μL) into a GC vial and BSTFA (150 μL) was added. The mixture was heated to 50 °C for 30 min. The silylated NBO-products were analyzed by GC-MS (Agilent 7890A GC-MS) equipped with aHP-5 capillary column (30 m × 250 μm) to identify the products by the comparison with the peak retention time and mass spectra of the authentic compounds. The identified products were quantified by GC-FID (Agilent 7890B) using the same column. Initial column temperature: 150°C (held for 10 min), raised at 5 °C/min to 280 °C (held for 20 min).

In the reported lignin upgrading reaction over the Ru/Nb_2O_5 catalyst, the yield of C_7~C_9 hydrocarbons (2606 μmol/g lignin) is comparable with that obtained by using the NBO method (2741 μmol/g lignin).

Supplementary Note 2: Stability test with substrate (0.1 g)/catalyst (0.1 g) ratio.

The stability test was also conducted using substrate (0.1 g)/catalyst (0.1 g) ratio in four consecutive recycling runs (Supplementary Figure 4). It found that the activity of catalyst decreased after the 1st run but stayed stable in the subsequent three recycling runs, and interestingly, the selectivity to arenes remained unchanged during the recycling runs. This is probably due to the reduced contact between the catalyst and the substrate and thus the retention of lignin monomers on the catalyst surface. When using substrate (0.1 g)/catalyst (0.2 g) ratio (Supplementary Figure 3), the catalyst remained high activity for all four runs.

Supplementary Note 3: *In situ* variable-temperature surface reaction.

In situ surface reactions of phenol conversion were conducted at 150, 170 and 185 °C over the Ru/Nb_2O_5 catalyst to study the temperature-dependence on the reaction selectivity. MS signals of benzene (*m/z* = 78) and cyclohexane (*m/z* = 84) were monitored continuously (Supplementary Figure 9), and the ratio of benzene/cyclohexane summarised in Supplementary Table 10. As the temperature ramped from 150 to 185 °C, the production of cyclohexane is constant, but the benzene/cyclohexane ratio increased rapidly, from 2.5 to 9.4. This result suggests that the production of benzene is strongly depending on the reaction temperature, indicating that the C–O bond cleavage is a kinetically-controlled process and high temperature favors the cleavage of $C_{aromatic}$–OH bonds to generate arenes selectively.

Supplementary Note 4: Inelastic neutron scattering (INS) spectral range.

The spectral range on TOSCA is 0 – 8000 cm^{-1}; however, the useful range is 0-2000 cm^{-1}. The reasons for this are explained in detail in a reported literature[7]. In brief, the scattered intensity is proportional to Q^{2n} (Q = momentum transfer, n = 1 for a fundamental, 2 for a first overtone or binary combination, etc…) multiplied by the Debye –Waller factor which depends on Q^2. On TOSCA there is a fixed relationship between energy transfer (Et) and momentum transfer: Et = 16.7 Q^2. Hence high energy transfer (e.g. 3200 cm^{-1} for the O-H stretch) means large momentum transfer. Thus large Q has two detrimental effects: it suppresses the total intensity via the Debye-Waller factor and, worse, the intensity of the overtones and combinations is proportional to Q^4, Q^6… for n = 2, 3… and their intensity relative to the fundamental makes the latter unobservable.

Supplementary Methods

Materials. The chemicals were purchased from commercial suppliers and used as provided: 4-methylphenol (J&K Chemical, 99%), 4-methylguaiacol (J&K Chemical, 99%), phenol (J&K Chemical, 99%), propylbenzene (TCI, >99% GC assay), ethylbenzene (TCI, >99% GC assay), 4-methylcyclohexanol (TCI, >98% GC assay), 4-methylcyclohexanone (TCI, >98% GC assay), methylcyclohexane (Macklin, ≥99.8% GC assay), ethylcyclohexane (J&K Chemical, 99%), propylcyclohexane (TCI, >98% GC assay). HZSM-5, ZrO_2, TiO_2 and Al_2O_3 were obtained from The Catalyst Plant of Nankai University. Activated carbon was purchased from SCM Industrial Chemical Co., Ltd. Birch wood, beech wood and pine wood were purchased from local manufactory (*ca.* 40 mesh), and dried at 100 °C for 5 h before use. All other chemicals were purchased from Sinopharm Chemical Reagent Co., Ltd. and used without purification.

Characterisation. Powder X-ray diffraction (PXRD) patterns were recorded on a Rigaku D/max-2550VB/PC using Cu Kα radiation (λ = 1.5406 Å). Transmission electron microscopy (TEM) images were recorded on a FEI Tecnai F20 s-TWIN instrument, and the electron beam accelerating voltage was at 200 kV. The nitrogen adsorption isotherms were performed on a Micromeritics ASAP 2020M sorption analyser. Prior to the measurement, the samples were outgassed at 200 °C for 8 h to remove trace amount of water from pores. The detailed characterizations of different support materials and supported Ru catalysts were shown in the Supplementary Information (Supplementary Figures 6, 7 and Supplementary Table 8).

Simulation. DFT calculations of the INS spectra for solid phenol, benzene, cyclohexane and cyclohexanol were carried out, which can be directly related to the experimental INS spectra of the solid state compounds with no approximations other than the use of DFT eigenvectors and eigenvalues to determine the spectral intensities. The information was used to identify the modes of vibrational features in the experimental INS spectra. No abscissa scale factor was used throughout this report for INS calculations. The calculated INS spectrum shows the total transitions (up to 10 orders). DFT calculations of the energies for surface adsorption and for the cleavage of C-O bonds were performed using the Vienna *Ab initio* Simulation Package (VASP)[1]. The calculations used the Projector Augmented Wave (PAW) method[2,3] to describe the effects of core electrons, and the Perdew-Burke-Ernzerhof (PBE)[4] implementation of the Generalized Gradient Approximation (GGA) for the exchange-correlation functional. The initial surface structures were generated by cleaving the corresponding surface in the crystal structure, and creating a vacuum slab of at least 1.5 nm. Then the atoms at the bottom layer were fixed while the other atoms were allowed to relax to their local potential energy minimum. The energy cutoff for the plane-waves is 500 eV, the energy tolerance for

electronic structure calculation is 10^{-4} eV, and the maximum force is below 0.01 eV/Å after structural relaxation.

Supplementary References

1. Kresse, G. and Furthmüller, J. Efficient iterative schemes for *ab initio* total-energy calculations using a plane-wave basis set. *Phys. Rev. B*, 54:11169 (1996).
2. Blochl, P. E. Projector augmented-wave method. *Phys. Rev. B*, 50:17953 (1994).
3. Kresse, G. and Joubert, D. From ultrasoft pseudopotentials to the projector augmented-wave method. *Phys. Rev. B*, 59:1758 (1999).
4. Perdew, J. P., Burke K. and Ernzerhof, M. Generalized gradient approximation made simple. *Phys. Rev. Lett.*, **77**, 3865-3868 (1996).
5. Shuai, L. *et al.* Formaldehyde stabilization facilitates lignin monomer production during biomass depolymerization. *Science* **354**, 329-333 (2016).
6. Li, Y., Akiyama, T., Yokoyama, T. & Matsumoto, Y. NMR assignment for diaryl ether structures (4–O–5 structures) in pine wood lignin. *Biomacromolecules* **17**, 1921-1929 (2016).
7. Parker, S. F., & Lennon, D. Applications of neutron scattering to heterogeneous catalysis. *J. Phys.: Conference Series* **746**, 012066 (2016).
